# Epidemiology, Virulence and Antimicrobial Resistance of *Escherichia coli* Isolated from Small Brazilian Farms Producers of Raw Milk Fresh Cheese

**DOI:** 10.3390/microorganisms12081739

**Published:** 2024-08-22

**Authors:** Laryssa Freitas Ribeiro, Gabriel Augusto Marques Rossi, Rafael Akira Sato, Andressa de Souza Pollo, Marita Vedovelli Cardozo, Luiz Augusto do Amaral, John Morris Fairbrother

**Affiliations:** 1 Mário Palmério University Center (UniFucamp), Av. Brasil Oeste, 1900, Jardim Zenith, Monte Carmelo 38500-000, MG, Brazil; laryssaribeiro84@gmail.com; 2 Department of Veterinary Medicine, University of Vila Velha (UVV), Vila Velha 29102-920, ES, Brazil; gabriel.rossi@uvv.br; 3 State University of São Paulo (UNESP), Via de Acesso Professor Paulo Donato Castelane Castellane S/N—Vila Industrial, Jaboticabal 14884-900, SP, Brazil; jaspionvet04@gmail.com (R.A.S.); andressa.souza@unesp.br (A.d.S.P.); marita.vedovelli@unesp.br (M.V.C.);; 4 Département de Pathologie et Microbiologie, Faculté de Médecine Vétérinaire, Université de Montréal, Saint-Hyacinthe, 3200 rue Sicotte, Saint-Hyacinthe, QC J2S 2M2, Canada

**Keywords:** antimicrobial resistance, dairy, enterobacteria, food microbiology, foodborne pathogens

## Abstract

This study aimed to identify contamination sources in raw milk and cheese on small farms in Brazil by isolating *Escherichia coli* at various stages of milk production and cheese manufacturing. The study targeted EAEC, EIEC, ETEC, EPEC, STEC, and ExPEC pathotypes, characterizing isolates for the presence of virulence genes, phylogroups, antimicrobial susceptibility, and phylogenetic relationships using PFGE and MLST. The presence of antimicrobial resistance genes and serogroups was also determined. Three categories of *E. coli* were identified: pathogenic, commensal, and ceftriaxone-resistant (ESBL) strains. Pathogenic EPEC, STEC, and ExPEC isolates were detected in milk and cheese samples. Most isolates belonged to phylogroups A and B1 and were resistant to antimicrobials such as nalidixic acid, ampicillin, kanamycin, streptomycin, sulfisoxazole, and tetracycline. Genetic analysis revealed that *E. coli* with identical virulence genes were present at different stages within the same farm. The most frequently identified serogroup was O18, and MLST identified ST131 associated with pathogenic isolates. The study concluded that *E. coli* was present at multiple points in milk collection and cheese production, with significant phylogroups and high antimicrobial resistance. These findings highlight the public health risk posed by contamination in raw milk and fresh cheese, emphasizing the need to adopt hygienic practices to control these microorganisms.

## 1. Introduction

The occurrence of foodborne diseases is one of the major causes of economic burden in low- and middle-income countries, necessitating better characterization for improved public health and greater socioeconomic development in these countries [1]. In this context, *Escherichia coli* is one of the most important and frequent foodborne bacteria reported in outbreaks. This microorganism is associated with intestinal and extraintestinal infections, and the pathotypes causing diarrhea in humans are known as diarrheagenic *E. coli* (DEC). These strains are classified into six categories: classical enteropathogenic *E. coli* (EPEC), shigatoxigenic *E. coli* (STEC), enterotoxigenic *E. coli* (ETEC), enteroaggregative *E. coli* (EAEC), enteroinvasive *E. coli* (EIEC), and diffusely adherent *E. coli* (DAEC) [2]. Additionally, extra-intestinal pathogenic *E. coli* (ExPEC) is found in the intestinal microbiota but is usually harmless in the intestine and may cause disease in extraintestinal sites [3].

This bacterium can be transmitted through the consumption of milk and dairy products obtained under inadequate conditions of production and processing [4]. Consuming frescal cheese made from raw milk may pose a significant risk of *E. coli* transmission. This type of cheese, known for its high moisture content and soft texture, creates an ideal environment for bacterial growth. Without proper pasteurization, harmful bacteria present in the raw milk can survive and multiply [5].

Various strains of diarrheagenic *E. coli* can be found in the environment of dairy farms and in raw milk. These bacteria, including strains with virulence potential and antimicrobial resistance, may colonize different ecological niches in these environments and persist due to an ability to form biofilms [6,7], potentially putting consumers at risk. The risk associated with consuming these foods may be even higher when antimicrobial-resistant pathogens are present, due to the difficulty in treating such infections. Animal-derived foods, such as milk and dairy products, can serve as sources of antimicrobial-resistant pathogens, and these bacteria can disseminate in the production environment on dairy farms and in cheese production [8]. Therefore, epidemiological studies are essential to identify the sources of dairy contamination. These studies use bacteriological typing techniques to establish genetic relationships between isolates from various points in the dairy production chain, including dairy products, animals, handlers, utensils, and equipment [9].

Thus, this study aimed to identify contamination sources in raw milk and cheese on small farms in Brazil by isolating *E. coli* at various steps and sites of milk production and fresh cheese manufacturing. The study aimed to detect the EAEC, EIEC, ETEC, EPEC, STEC, and ExPEC pathotypes and to characterize the isolates by analyzing virulence genes, phylogroups, antimicrobial susceptibility, and genetic and epidemiological relationships using PFGE and MLST. Additionally, the research sought to investigate antimicrobial resistance genes and classify the isolates into serogroups.

## 2. Materials and Methods

### 2.1. Sampling

Samples were collected from five small dairy farms (named A, B, C, D, and E) located in the northeast of São Paulo State, Brazil. These farms produce fresh cheeses using raw milk. The collected samples included bovine feces; milkers’ hands; milking buckets; raw milk; artisanal raw milk cheeses; whey; water; cheese-processing surfaces; cheese handlers’ hands; and cheese sieves, trays, molds, and skimmers. A total of 106 samples were collected, with 22 samples from farm A, 23 from farm B, 21 from farm C, 21 from farm D, and 19 from farm E. On the day of collection, the milking and cheese production processes were monitored at each farm to ensure the accurate sampling and representation of the production environment.

### 2.2. Sample Preparation

The procedures of this step were performed according to Gill and colleagues [10]. Upon arrival at the laboratory, 100 mL aliquots of water samples were filtered through a sterile membrane filter (47 mm diameter, 0.45 μm porosity). The membranes were then cut with sterile scissors and placed in flasks containing 50 mL of trypticase soy broth (TSB) and incubated at 42 °C for 4 h.

For cheese samples, portions were combined into a 25 g representative sample and placed in a sterile bag with 225 mL of TSB. This mixture was homogenized using a Stomacher^®^ for 2 min, then transferred to flasks and incubated at 42 °C for 4 h. For cheese serum samples, 10 mL aliquots were transferred to flasks containing 90 mL of TSB, which were also incubated at 42 °C for 4 h. Similarly, 25 mL aliquots of milk were transferred to flasks containing 225 mL of TSB and incubated at 42 °C for 4 h.

Tubes containing swabs (including those with bovine feces) and peptone water (5 mL) were vortexed to ensure proper homogenization. Subsequently, 1 mL of the suspension was added to flasks containing 5 mL of TSB and incubated at 42 °C for 4 h. Following 4 h of incubation to allow bacterial multiplication, the antibiotics vancomycin (10 μg/mL) and cefsulodin (3 μg/mL) were added to vials containing water aliquots, cheese samples, and serum, as well as tubes with bacterial cultures from swabs. Incubation was continued at 42 °C for 16 to 20 h. After incubation, 700 μL aliquots were taken from each culture and frozen at −80 °C in brain heart infusion (BHI) broth with 20% glycerol for further analysis. These samples were also plated on nutrient agar. After incubation, the samples were transported to the *E. coli* Laboratory (EcL) of the Faculty of Veterinary Medicine at the Université de Montréal in Saint-Hyacinthe, Quebec, Canada, for phenotypic and genotypic analyses of the isolated *E. coli* strains.

### 2.3. E. coli Isolation

**Figure 1:**
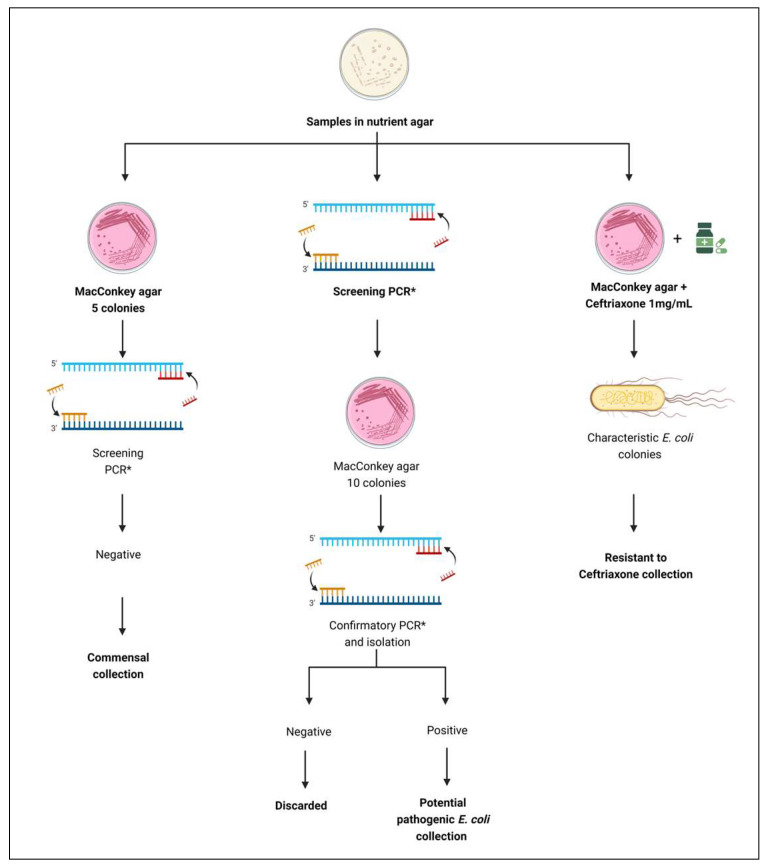
Flowchart for obtaining three collections of *E. coli* from various sources—water, bovine feces, milk, cheese, surfaces and utensils used in cheese production, and cheese handlers—across five small dairy farms producing raw milk cheese in the northeastern São Paulo State, Brazil. Isolates were considered to be potentially pathogenic if positive for the following virulence genes defining the STEC (*stxA*, *stx2A*), ETEC (*estA*, *estB*, *eltB*, *faeG*), ExPEC (*cnf*, *papC*, *iucD*, *tsh*, *sfa*, *afa*, *kpsM II*), EIEC (*ipaH*, *ial*), and EAEC (*aaiC*, *aatA*, *aggR*) pathotypes. Virulence genes defining pathogenic *E. coli* for EAEC, EIEC, ETEC, EPEC, STEC, and ExPEC categories.

Three distinct collections of *E. coli* were assembled: (1), commensal isolates, (2) potentially pathogenic isolates, and (3) ceftriaxone-resistant isolates. This process followed the flowchart depicted in Figure 1.

#### 2.3.1. Collection of Commensal *E. coli* Isolates

Bacterial cultures from nutrient agar were inoculated onto Petri dishes containing MacConkey agar and incubated at 37 °C for 24 h. After incubation, three colonies from each sample were selected (suggestive of *E. coli* but morphologically distinct), inoculated, and stored in a −80 °C freezer in a 30% glycerol solution. This collection excluded isolates positive for any of the tested virulence genes. After PCR analysis to identify these genes, any isolates found to possess them were eliminated from the collection.

#### 2.3.2. Collection of Potentially Pathogenic *E. coli* Isolates

To detect potentially pathogenic *E. coli* isolates, sample culture aliquots were inoculated into tubes containing BHI and incubated overnight at 37 °C. DNA extraction was then performed using the boiling method [11]. PCR was conducted to screen for the presence of virulence genes defining the pathotypes STEC (*stxA*, *stx2A*), ETEC (*estA*, *estB*, *eltB*, *faeG*), ExPEC (*cnf*, *papC*, *iucD*, *tsh*, *sfa*, *afa*, *kpsM II*), EIEC (*ipaH*, *ial*), and EAEC (*aaiC*, *aatA*, *aggR*) using primers described in Supplementary File S1 [12,13,14,15,16,17,18,19,20,21,22,23,24,25,26]. Each PCR amplification reaction included 1x buffer (20 mM Tris-HCl pH 8.4; 50 mM KCl), 2 mM MgCl2, 0.2 mM dNTPs, 2 U of Taq DNA polymerase, 4 pmol of each primer, 5 µL of genomic DNA, and sterile distilled water to reach a final volume of 25 µL. The reactions were performed in multiplex formats: one for *stxA*, *stx2A*, and *eae* genes; one for *bfp*; one for *estA*, *estB*, *eltB*, and *faeG* genes; one for *cnf*, *papC*, *iucD*, and *tsh* genes; one for *sfa* and *afa* genes; one for *kpsMII*; one for *ipaH* and *ial* genes; and one for *aaiC*, *aatA*, and *aggR* genes. The PCR cycles were carried out in a thermocycler with the following program: initial denaturation at 94 °C for 5 min, followed by 30 cycles of 94 °C for 30 s (denaturation), gene-specific annealing temperature for 30 s, and 72 °C for 30 s (extension). The final cycle included an extension at 72 °C for 10 min to ensure complete extension by the Taq DNA polymerase. A negative control containing only water, without DNA, was included in each reaction batch.

Confirmation of the presence of positive colonies in samples positive for any of the tested genes involved seeding them on MacConkey agar plates, followed by incubation at 37 °C for 24 h. Subsequently, ten to fifteen characteristic *E. coli* colonies from each plate were transferred onto a single Petri plate containing MacConkey agar. After incubation, these colonies were re-inoculated in tubes containing BHI broth to facilitate further bacterial growth for DNA extraction by boiling. This DNA pool was then tested for the presence of virulence genes. If the pool tested positive for any of the target genes, the individual colony from the positive pool was seeded in BHI broth to prepare the DNA template, followed by PCR to confirm the presence of virulence genes in the isolate. If the positive isolate was not found among the first 10 colonies tested, the process was repeated by seeding another 10 or 15 characteristic colonies onto a MacConkey plate until the positive isolate(s) was identified.

#### 2.3.3. Collection of Ceftriaxone-Resistant *E. coli* Isolates

To collect *E. coli* isolates resistant to ceftriaxone, that is, extended-spectrum β-lactamase/AmpC- β-lactamase (ESBL/AmpC)-producing isolates, bacterial cultures from nutrient agar were inoculated into tubes containing 10 mL of 0.1% peptone water with ceftriaxone and incubated at 37 °C for 30 min. This culture was then plated onto MacConkey agar supplemented with ceftriaxone (final concentration of 1 mg/L) using sterile swabs. After the medium surface dried, the plates were inverted and incubated at 37 °C for 24 h. For analysis, a positive control of *E. coli* resistant to ceftriaxone (CRO-AMR-133.1) and a negative control sensitive to ceftriaxone (GEN-AMR-127.2) were included. Characteristic *E. coli* colonies were selected for the ceftriaxone-resistant collection. Additionally, isolates from the ceftriaxone-resistant collection underwent PCR testing to detect genes associated with STEC, EPEC, ETEC, EIEC, EAEC, and ExPEC pathotypes (Supplementary File S1).

### 2.4. Phylogroup

**Figure 2:**
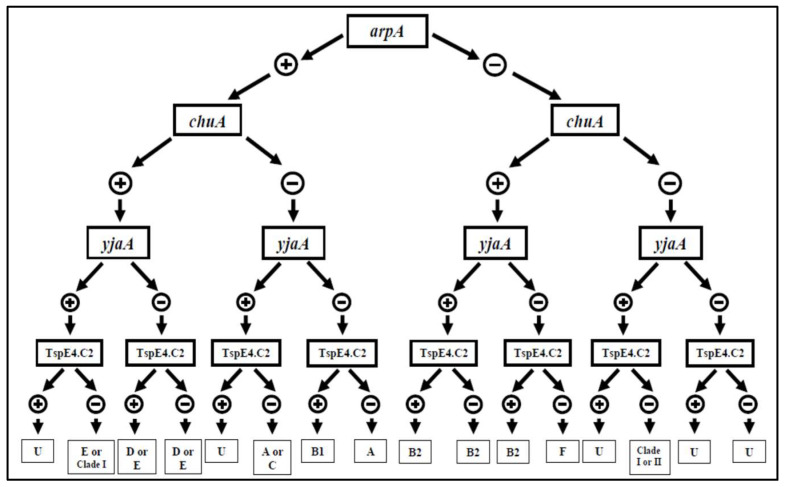
The dichotomous tree used to determine the phylogenetic group of *E. coli* strains based on PCR results for the genes *chuA*, *YjaA*, *TspE4C2*, *Acek / ArpA1*, *ArpAgpE*, *trpAgpC*, and *trpBA* [27].

For isolates of all three collections, the presence *of chuA*, *YjaA*, *TspE4C2*, *Acek/ArpA1*, *ArpAgpE*, *trpAgpC*, and *trpBA* genes was identified using the initiator oligonucleotides outlined in Supplementary File S2 [27]. This process followed the dichotomous tree structure illustrated in Figure 2.

### 2.5. Antimicrobial Susceptibility Test

All isolates from the three *E. coli* collections underwent antimicrobial susceptibility testing according to the method outlined by Bauer et al. (1966) [28]. *E. coli* ATCC 25922 was used as the control strain. Plates were then incubated at 37 °C for 24 h.

The antimicrobials tested included amoxicillin/clavulonic acid (30 μg), ampicillin (10 μg), cefoxitin (30 μg), ceftriaxone (30 μg), chloramphenicol (30 μg), ciprofloxacin (5 μg), gentamicin (10 μg), gentamicin (10 μg) kanamycin (30 μg), nalidixic acid (30 μg), streptomycin (10 μg), sulfisoxazole (250 μg), tetracycline (30 μg), trimethoprim/sulfamethoxazole (23.75 μg), and ceftiofur (30 μg). The inhibition zones were compared with those established by the Clinical and Laboratory Standards Institute [29].

### 2.6. Epidemiological Analysis of E. coli Isolates Using Pulsed-Field Gel Electrophoresis (PFGE)

A set of 172 isolates was chosen from three collections of *E. coli*. One isolate was selected from each sample of each farm, ensuring representation across various sources (e.g., water, milk, serum). Additionally, isolates were chosen from each previously established phylogroup. In cases where several isolates from the same sample belonged to different phylogroups, all were selected. Furthermore, isolates were chosen based on their resistance profiles, with priority given to those resistant to the highest number of antimicrobials. Thus, a total of 134 isolates were selected from the commensal collection, with 26 from farm A, 28 from farm B, 26 from farm C, 29 from farm D, and 25 from farm E. Additionally, 37 isolates were chosen from the potentially pathogenic collection, and one isolate was selected from the ceftriaxone-resistant collection from farm C.

PFGE was conducted following the methodology outlined as described by Ribot et al. (2006) [30], using the CHEF DR III system at 14 °C in TBE 0.5× buffer. DNA cleavage was achieved using 0.2–0.8 U *XbaI* restriction enzyme, according to the manufacturer’s instructions. Pulsed-field electrophoresis was performed for 18 h, ranging from 2.2 to 54 s at 6 V. Gel band profiles were analyzed using Bionumerics software (Applied Maths, Kortrijk, Belgium, version 6.6.11), which generated a dendrogram at the conclusion of the analysis. The molecular size standard employed was *Salmonella* serovar Braenderup H9812, as described by Ribot et al. (2006) [30], providing known band sizes for reference.

### 2.7. Molecular Analysis for Identification of Resistance Genes by PCR

*E. coli* isolates chosen for PFGE analysis, exhibiting in vitro resistance to antimicrobials from the β-lactam (blaSHV, blaTEM, blaCMY-2, blaOXA-1, blaCTX-M), tetracycline (tetA, tetB, tetC), nalidixic acid and ciprofloxacin (qnrB), streptomycin (aadA1), trimethoprim/sulfamethoxazole, and sulfisoxazole (dhfI, dhfrV, dhfrVII) groups, underwent examination for the presence of genes associated with resistance using multiplex PCR (Supplementary File S3) [31,32,33,34,35,36]. Uniplex reactions were conducted with primers for *aadA*, *tetA*, *tetB*, *tetC*, *dhfr I*, *dhfr V*, *dhfr VII*, and *qnrB*, while a multiplex reaction was performed for *blaCMY-2*, *blaTEM*, *blaSHV*, *blaOXA*, and *blaCTX-M*.

### 2.8. Serotyping for Detection of Somatic Antigen (O)

Eighteen isolates were selected from the potentially pathogenic collection based on genetic representativeness according to PFGE for the detection of somatic antigen (O). Isolates were cultured on TSA agar plates and maintained at 37 °C for 24 h. Subsequently, 1 mL of sterile phosphate-buffered saline (PBS) was added to the bacterial culture, followed by homogenization. After autoclaving, the bacterial suspension was tested with antisera provided by the ECL of the University of Montreal (Supplementary File S4). The presence of agglutination confirmed using an agglutinoscope, indicated positive results, enabling the identification of the somatic antigen of the isolate.

### 2.9. Multilocus Sequence Typing (MLST)

The 18 isolates chosen for serotyping underwent sequencing of PCR products amplified from the *adk*, *fumC*, *gyrB*, *icd*, *mdh*, *purA*, and *recA* genes (Supplementary File S5), following the protocol outlined earlier. PCR products were purified using the QIAquick PCR Purification Kit according to the manufacturer’s guidelines. Sequencing was performed at the Diagnostic Service—Faculté de médecine vétérinaire de l’Université de Montréal, utilizing the BigDye^®^ Terminator kit in an ABI PRISM 3500 DNA Analyzer sequencer. Quality evaluation, consensus sequence generation, and trimming were conducted using the Phred/Phrap/Consed software package [37]. Sequences were filtered to ensure a minimum phred quality of 20 or higher. Next, these sequences were compared against the GenBank database using the BLAST tool. Subsequently, they were aligned with sequences in the MLST database to identify the sequence type (ST).

For phylogenetic analysis, sequences of each gene and those in the database were aligned separately using MUSCLE software available in MEGA 6.06 software [38]. Sequences of *E. coli* O157 and *Salmonella enterica* subsp. enterica were utilized as reference sequences. Following alignment, sequences were concatenated and evaluated to determine the most appropriate evolutionary model based on the Akaike information criterion (AIC). The phylogenetic tree was constructed using MrBayes 3.2.3 software with substitution type six and distribution I + G, employing the Markov Chain Monte Carlo algorithm. Four independent runs were conducted with 10,000,000 generations, sampled every 100 generations. After analysis, trees with a standard deviation equal to or less than 0.01 were retained, discarding 25% of trees as burn-in. Finally, the resulting phylogenetic tree was graphically edited using TreeGraph 2.3.0 software.

## 3. Results and Discussion

### 3.1. Collections of E. coli Isolates

#### 3.1.1. Commensal

Three commensal isolates were obtained from each sample, totaling 303 isolates: 66 from farm A, 60 from farm B, 57 from farm C, 63 from farm D, and 57 from farm E. The presence of *E. coli* in all samples likely resulted from poor hygiene practices during milking and cheese production. For example, farms A, C, D, and E did not clean their ceilings properly before milking, and farm B used only water for cleaning, lacking proper sanitation procedures. Additionally, traces of milk from previous milking sessions were found on utensils such as sieves across all farms, indicating inadequate washing. Milk and dairy products are rich in essential nutrients, such as proteins, fats, carbohydrates, vitamins, and minerals, which support microbial growth. Therefore, the combination of poor hygiene practices and nutrient-rich environments likely contributed to the presence and proliferation of *E. coli* in the samples.

#### 3.1.2. Potentially Pathogenic

Out of 106 samples collected from five farms, 29 (27.36%) demonstrated the presence of potentially pathogenic *E. coli* strains (Supplementary File S6). The highest frequency was observed in farms A and C, with 36.36% (8 out of 22 samples) and 42.86% (9 out of 21 samples) positivity, respectively. Farms A and C had uncovered and unpaved pens, leading to higher levels of bovine fecal contamination during milking. Additionally, the water-filtering membranes in these farms appeared visually dirtier compared to others.

The predominant pathotype found on farm A was STEC, coinciding with its high contamination level due to the high amount of bovine feces [39]. Farm C sourced water from both mines and rivers, resulting in the visually dirtiest filtering membrane among all farms and the highest number of samples positive for ExPEC. ExPEC is part of the intestinal microbiota of various animal species and is found in environmental sources such as surface water, rainwater, and wastewater [40].

Only two out of the six pathotypes of diarrheal *E. coli*—STEC and EPEC, as well as ExPEC—were identified in the samples. Defining virulence genes were detected in samples of water, milk, molds, and fresh cheese. Other studies on *E. coli* prevalence in milk and cheese have also reported potentially pathogenic strains. For example, one study found DEC and ExPEC in 36.9% of 72 raw milk samples, 55 “Karish” cheese samples, and 60 “Ras” cheese samples, with 46.8% of 222 *E. coli* isolates harboring one or more virulence gene [41]. These findings align with our study, identifying ExPEC, STEC, and EPEC as the most prevalent pathotypes. The presence of STEC in cheese samples from our study, as well as in raw milk and cheese, can be attributed to fecal contamination from cattle [39].

Virulence gene-positive isolates were found in all but 6 of the 29 positive samples. This challenge arose because strains carrying virulence genes may exist in low concentrations in samples, making it difficult to isolate specific cells. This scenario occurred in water and bovine feces samples from farm A and in samples from bovine feces, buckets, and a spoon on farm B and water in the cheese-making area on farm D. Nevertheless, at least one potentially pathogenic isolate was successfully found in all other samples. Consequently, a set of 73 potentially pathogenic *E. coli* isolates were obtained: 18 from farm A, 18 from farm B, 29 from farm C, 5 from farm D, and 3 from farm E (Supplementary File S7). Most of these isolates belonged to the ExPEC pathotype, frequently encountered in animal-derived foods, highlighting their potential to vehicle foodborne pathogens [42].

Notably, isolates carrying the *stx2* gene were found in both bovine feces and milk samples on farm A. Potentially pathogenic ExPEC samples containing the *kps* gene were detected in bovine feces, liners, and water from the cheese-making environment on farm B. Although there were only a few contaminated samples in farm B, a total of 18 such isolates were observed, which was a higher count compared to other farms. These isolates were predominantly found in the liners and may be attributed to the inadequate washing of milking equipment.

On farm C, the *iuc*D gene was detected in isolates from the milk collection bucket, strainer, cheese-making surface, mold, and cheese whey. Farms D and E exhibited the circulation of the *kps* gene between bovine feces and milk samples. These analyses highlighted the presence of potentially pathogenic *E. coli* strains in milk collection and cheese production, emphasizing the need for enhanced hygiene protocols. Potentially pathogenic strains from the same farm typically exhibit consistent virulence gene profiles due to the horizontal transfer of virulence genes among *E. coli* cells [43]. This poses a significant public health risk, as these microorganisms can transfer their virulence genes to commensal strains, making them potentially pathogenic and increasing the prevalence of harmful strains in raw milk fresh cheese.

#### 3.1.3. Presence of ESBL/AmpC-Producing Isolates

Among the 106 samples examined, only 1 sample of bovine feces from farm C exhibited resistance to ceftriaxone. From this sample, five resistant isolates were obtained. Notably, these isolates did not test positive for any of the assessed virulence genes. The presence of ceftriaxone-resistant bacteria in animals used for food production, such as meat and milk, may be due to the use of third- and fourth-generation cephalosporins in farm animals [44].

In the case of farm C, the detection of ceftriaxone-resistant isolates in bovine feces may be linked to the inappropriate use of antimicrobials. The owner reported administering antibiotics until the animal had health improvement, without following the recommended duration of treatment. This misuse of antimicrobial agents in farm animals has led to the emergence of resistant microorganisms and has reduced the efficacy of these drugs in therapeutic applications. The global rise in resistance to ceftriaxone presents a significant challenge. Consequently, food can serve as a crucial vehicle for the dissemination of antimicrobial-resistant *E. coli* [42,45].

### 3.2. Phylogrouping

In the collection of commensal isolates, samples from farm A were predominantly classified into the phylogroup B1, while isolates from other farms were mostly assigned either to group A or B1. In the potentially pathogenic isolate collection, farms A and C also exhibited isolates mainly falling into phylogroup B1. Conversely, farm B showed isolates spanning four phylogroups, B2, D, E, and F; coincidentally, this was the only farm employing mechanical milking and where observations revealed a lack of dairy line cleaning. Farm D presented isolates distributed across phylogroups A, B1, and predominantly group D. The ESBL/AmpC-producing isolates from farm C exclusively classified into group B1 (Supplementary File S8).

In our study, phylogroups A and B1 were the most prevalent, consistent with findings from a previous investigation [46]. The authors examined the antibiotic resistance profiles and virulence factors of *E. coli* strains from wastewater and human commensal isolates. It was noted that phylogroups A and B1 were frequently found in these environments, with B1 isolates showing significant resistance to multiple antibiotics. The study emphasized the adaptability of these phylogroups to different environmental conditions, contributing to their widespread distribution. Group A strains are frequently associated with humans, cattle, and pigs, whereas those in group B1 are commonly found in cattle and sheep, and groups B2 and D are predominantly associated with humans [47]. ExPEC often belongs to the B2 and D phylogroups [48], which were the most common in the farm B potentially pathogenic collection.

As observed with *E. coli* virulence genes, isolates belonging to the same phylogroup were found across different stages of milk and cheese production (Supplementary File S9). In farm A, isolates from bovine feces, buckets, and milk exclusively belonged to phylogroup B1, despite harboring different virulence genes. In farm B, isolates from liners and water in the cheese-making room were categorized under phylogroup B2. Farm C exhibited isolates from group B1 circulating among samples from buckets, sieves, cheese-making surfaces, molds, and cheese whey. In farm D, isolates from bovine feces and milk belonged to phylogroup D. Meanwhile, farm E had isolates from milk and a sieve categorized as phylogroup A. These findings suggest that cross-contamination between milk and cheese production stages is occurring across all five farms analyzed. Additionally, failures in management and hygiene were evident across all investigated stages.

Most of the potentially pathogenic *E. coli* isolates belonging to groups A, B2, D, or F are categorized as ExPEC. This trend was observed in chickens in Canada [49] and also in a study conducted in a hospital in Paraná, Brazil [50]. The authors analyzed urine and blood samples from patients and linked ExPEC isolates to phylogenetic group B2, whereas commensal isolates were more commonly associated with group A [50]. Moreover, a study highlighted the significant presence of phylogroup B2 *E. coli* in various environmental sources, including surface water, sewage, and rainwater. These sources were found to contain *E. coli* strains that are genetically similar to human-associated ExPEC, suggesting a potential route for the transmission of these pathogens from animals to humans through environmental exposure [51]. Furthermore, isolates from phylogroup B2 harbor a greater number of virulence genes [52].

These isolates with heightened pathogenic potential were discovered in water samples from the cheese-making and milking lines in farm B, as well as in milk samples from farm D. The presence of such microorganisms in milk and cheese production, particularly when using raw milk, underscores the potential risk of contamination in the food and the subsequent threat to public health.

### 3.3. Antimicrobial Sensitivity Test

The antimicrobial sensitivity test revealed resistance among *E. coli* isolates from all three collections (Supplementary File S10). In the commensal isolate collection, approximately 35.31% of the isolates exhibited resistance to at least one antimicrobial agent. Notably, farm B demonstrated resistance to the highest number of antimicrobials, with 12 out of the 14 tested showing resistance. Across all sampled farms, isolates commonly displayed resistance to nalidixic acid, a significant concern for public health, followed by ampicillin, an important medication in human medicine, and tetracycline, frequently used in dairy herds, particularly for treating persistent mastitis. It is noteworthy that nalidixic acid is not approved for veterinary use in Brazil. Therefore, it is intriguing to observe resistance to this antimicrobial among the isolates. However, other fluoroquinolones such as ciprofloxacin and enrofloxacin are used in cattle in Brazil. Resistance to nalidixic acid is always observed before resistance to ciprofloxacin following the treatment of animals with fluoroquinolones. This serves as a warning that ciprofloxacin resistance due to the use of fluoroquinolones in animals is imminent [53].

The potentially pathogenic isolates exhibited resistance to at least one of ten antimicrobials, with 69.8% of the population being resistant. This notable resistance, predominantly observed in potentially pathogenic strains, could be attributed to the presence of animals with clinical mastitis across all visited farms, likely stemming from the indiscriminate or inappropriate use of antimicrobials. Notably, in farm C, where all five animals had clinical mastitis, 93% of the isolates were resistant to ampicillin, streptomycin, sulfisoxazole, and trimethoprim/sulfamethoxazole. In farm B, 77% of the isolates showed resistance to nalidixic acid and tetracycline. These findings underscore a concerningly high prevalence of antimicrobial-resistant isolates, which holds significant implications for human therapy. Additionally, the presence of virulence genes in *E. coli* has been linked to antimicrobial resistance [54,55]. This observation supports the notion that potentially pathogenic strains tend to harbor a greater proportion of antimicrobial-resistant isolates.

A study conducted in Iran examined 120 potentially pathogenic *E. coli* isolates from 200 raw milk samples and 50 cheeses made from unpasteurized milk. The findings revealed alarming levels of antimicrobial resistance, with all isolates showing resistance to oxytetracycline, 86% to cephalexin, 56% to nalidixic acid, 42% to nitrofurantoin, 30% to gentamicin, and 28% to trimethoprim/sulfamethoxazole (Bonyadian, Moshtaghi, Taheri, 2014). The authors cautioned that these pathogenic and resistant *E. coli* strains present in raw milk and cheese pose a significant risk of transferring resistance factors to consumers’ intestinal microbiota [54].

Interestingly, the isolates in our study also exhibited resistance to the antimicrobials mentioned above. Another study conducted in Turkey analyzed 146 *E. coli* isolates from human urine and found resistance to a range of antimicrobials including cefazolin, ampicillin, nalidixic acid, ciprofloxacin, norfloxacin, tetracycline, trimethoprim/sulfamethoxazole, amoxicillin, and ceftriaxone [56]. Remarkably, the resistance profile observed in the *E. coli* isolates from human urine mirrors that of the isolates in our study, spanning both commensal and potentially pathogenic collections.

The isolates from the ESBL/AmpC producer collection exhibited 100% resistance to amoxicillin, ampicillin, ceftiofur, and cefotixin. A study conducted in Germany comparing *E. coli* isolates from dairy farms and beef cattle farms found a higher prevalence of ESBL isolates on dairy farms, which displayed resistance to ampicillin, cefazolin, cefuroxime, cephalexin, cefotaxime, and trimethoprim/sulfamethoxazole [57]. The isolates in our study’s ESBL/AmpC collection were sensitive to ceftriaxone and resistant to ceftiofur, indicating they are not ESBL but rather AmpC, possibly due to the treatment of animals with ceftiofur. This is consistent with the resistance to cefoxitin that we observed. Therefore, we found no evidence of ESBLs, which is encouraging. However, the presence of AmpC is a warning about the use of ceftiofur in cattle.

### 3.4. Epidemiological Analysis of E. coli Isolates Using Pulsed-Field Electrophoresis Gel (PFGE)

The PFGE analysis was conducted on 172 *E. coli* isolates from three collections to explore genetic similarities among isolates from various milk and cheese production points. The resulting dendrograms (Supplementary File S11) revealed 41 clusters, some grouped based on isolate origin, farm, or sample source, as well as similarities in phylogroups and the presence of identical virulence genes. Notably, genetic similarities were also observed among isolates from different farms and samples.

In the dendrogram, it became evident that isolates from farm B, carrying the *kps* gene and falling into phylogenetic group B2, derived from samples of liners and water from the cheese-making room, displayed significant genetic resemblance. Similar patterns were observed with isolates from farm C, belonging to phylogenetic group B1, sourced from buckets, sieves, cheese surfaces, cheese whey, molds, and cheese. All isolates exhibiting genetic likeness from farm C and phylogenetic group B1 demonstrated the presence of the *iucD* gene, except for the cheese isolate; nevertheless, they all clustered together. This is an MDR clonal lineage and may have spread throughout different sites on the farm.

Furthermore, isolates from farm E, belonging to phylogroup A and sourced from bucket and sieve samples, exhibited substantial genetic similarity among themselves, as did isolates from bovine feces and water samples from the cheese-making room. These findings provide further evidence of *E. coli* cross-contamination, including potentially pathogenic MDR strains, across various stages of cheese and milk production within the surveyed farms. This underscores the imperative for implementing good dairy obtainment and cheese manufacturing practices across all farms, encompassing a suite of measures to ensure the sanitary quality and safety of dairy products.

A study examining the significance of STEC in dairy production highlighted various pathways through which milk and dairy products can become contaminated. These include cross-contamination between different animal species, exposure to bovine feces, effluents, animal feed, and contaminated water sources [58]. Moreover, additional research suggests that environmental factors, as well as improper handling and processing of food by untrained individuals, contribute to contamination risks [59]. Indeed, food products prepared through homemade processes are particularly susceptible to contamination due to the use of raw materials from unsafe sources and inadequate sanitation practices [60].

Studies employing pulsed-field gel electrophoresis (PFGE) to explore genetic diversity have revealed similarities among *E. coli* isolates obtained from various sources, including raw milk and cheeses [41]. Furthermore, PFGE has been used to examine genetic resemblances among *E. coli* isolates from urinary tract infections (UTIs) in women, meat, and ready-to-eat foods served in restaurants. These investigations revealed associations between *E. coli* from chicken meat and other food items and those recovered from UTIs [61]. Similarly, potentially pathogenic *E. coli* strains identified in milk and cheese samples may be directly linked to human infections.

### 3.5. PCR for Detecting Genes Related to Antimicrobial Resistance

Most antimicrobial-resistant isolates exhibited gene amplification associated with resistance during in vitro testing. Within the β-lactam group, *blaTEM* was the most prevalent gene. The *aadA1* gene was detected across all farms, with a higher occurrence among isolates from the commensal collection from farms A, C, and D. Notably, only isolates classified as ExPEC or potentially ExPEC carried the *blaTEM* gene, while the *aadA1* gene was absent in these isolates (Supplementary File S12). None of the isolates in the commensal and pathogenic collections were positive for CTX-M or CMY.

A study examined resistance genes in *E. coli* isolates from raw milk in Egypt. Out of 450 milk samples collected from both healthy and mastitic cows and buffaloes, the authors found that *E. coli* was isolated from 33 mastitic milk samples (9.1%). Notably, the *bla*TEM gene was detected in 94% of the *E. coli* isolates from mastitic milk, indicating a high prevalence of this resistance gene among the isolates [62]. In this context, a study investigated the resistome of high-risk pandemic clones of multidrug-resistant extra-intestinal pathogenic *E. coli* (ExPEC) in Uganda. The authors highlighted the presence of the *aadA1* gene among other resistance genes, demonstrating its widespread occurrence in healthcare-associated infections. The study emphasized the role of horizontal gene transfer in spreading resistance genes like *aadA1* among bacterial populations in clinical settings [63]. Consequently, the mere presence of *E. coli* isolates carrying these genes in milk production and cheese manufacturing poses a significant health risk to consumers.

In the case of isolates in the ESBL/AmpC collection, all of them were found to possess the *blaCMY* gene and none were positive for CTX-M. A study conducted in Germany, comparing *E. coli* isolates from dairy and beef cattle farms, also detected the presence of this gene in ESBL isolates, albeit in a smaller proportion, accounting for only 2.55% (5 out of 196 isolates), marking the first report of ESBL in the country [57]. This occurrence could likely be attributed to the widespread use of third- and fourth-generation cephalosporins in livestock [44].

Concerning the genes associated with tetracycline resistance, *tetA* was identified as the most prevalent, followed by *tetB* and *tetC*. Notably, the latter two genes were absent in potentially pathogenic isolates, and most were negative for *tetA*, suggesting the involvement of another *tet* gene (Supplementary File S13). A study examining the prevalence of virulence and antimicrobial resistance genes in *E. coli* isolates from dairy and beef cattle in Poland found the *tetA* gene in 25% of the isolates, while both *tetB* and *tetC* were present in 2.9% of the isolates, exclusively from dairy cattle [64]. These findings may be linked to the frequent use of antimicrobials in dairy cattle, particularly for treating mastitis, which promotes the selection of resistant bacterial strains [65]. In our study, a higher prevalence of the *tetA* gene was observed compared to the findings.

Another study focused on multidrug-resistant *E. coli* strains isolated from environmental, animal, and human samples in Panama. The study found that 45% of the samples contained either the *tetA* or *tetB* genes, or both. Specifically, *tetA* was more prevalent, detected alone or in combination with *tetB* in various sources such as chicken, swine, and cow samples [66].

The *qnrB* gene, responsible for conferring resistance to nalidixic acid and ciprofloxacin, was notably prevalent among isolates from all three collections, being identified in 100% of the potentially ExPEC isolates. However, in the collection of commensal isolates, this gene was absent in farm E. A study analyzed *E. coli* isolates carrying the *qnrB* gene and it was found that this gene was associated with plasmidic sequences in these isolates. The study identified multiple plasmid incompatibility groups, indicating that *qnrB* is frequently located on diverse plasmids, facilitating its spread among bacterial populations. The authors underscore the significant role of plasmids in the dissemination of quinolone-resistance genes like *qnrB* [67].

Furthermore, *dfr1* was detected in only three isolates from the commensal collection (two from Farm A and one from Farm B) and was not detected in any other collections. The *dfr5* gene was found in three isolates from the commensal collection (from farms A, C, and E) and in ten isolates classified as ExPec in the potentially pathogenic collection (Supplementary File S13). These results suggest that other *dfr* gene variants might be involved, which were not tested in our study.

### 3.6. Serological Testing for the Detection of Somatic Antigen (O)

Among the 18 *E. coli* isolates from the potentially pathogenic collection analyzed, 12 different serogroups were identified. Serogroup O18 was the most prevalent, accounting for 27.8% of the isolates (5 out of 18), and was found in farms A, B, C, and E (Supplementary File S14). Interestingly, serogroup O18 was exclusively present in ExPEC or potential ExPEC isolates, spanning different phylogroups. These isolates were recovered from various sources such as buckets, sieves, water from the cheese-making room, the inner surface of liners, and cheese samples. Additionally, serogroups O138 and O126, identified in milk and bovine feces samples from farms A and D, respectively, are known to be significant pathogens in human infections.

A study conducted on *E. coli* isolates associated with neonatal meningitis revealed that 13.2% of these strains belonged to serogroup O18 [68]. NGUYEN and colleagues highlighted that serogroup O18 strains are significant in both neonatal meningitis and UTIs, emphasizing the presence of virulence factors and resistance genes in these isolates [69]. These findings underscore the significant role of serogroup O18 in causing serious human infections, posing a potential public health risk not only because these strains are present in the production chain for cheese originating from raw milk but also because they are present in the cheese itself. Furthermore, a case of uremic hemolytic syndrome associated with Shigatoxin-producing *E. coli* of serogroup O138 has been reported [70]. This serogroup was also detected in the current study in milk samples, highlighting the considerable risk of contamination in the final product, cheese originating from raw milk. Serogroup O126, also identified in this study, is commonly associated with urinary tract infections [71].

### 3.7. Multilocus Sequence Typing (MLST)

Based on the MLST analysis, the isolates were categorized by sequence type (ST) (Supplementary File S15). Generally, there was no apparent correlation between the ST and the source of the isolates; for instance, isolates 2B-7 and 4D3-6, both with ST38, originated from different farms and sample types and are of different O groups.

The presence of ST131 in this study is noteworthy. ExPEC strains of ST131 have been identified in chicken meat and linked to human UTIs [61]. Moreover, this ST has been associated with urinary tract infections in children [72] and systemic infections in Korea [73]. The infectivity, transmissibility, and pathogenicity of strains of this ST have been highlighted, suggesting their potential endemicity worldwide [74]. Additionally, data from studies analyzing the genotypic and phenotypic profiles of *E. coli* ST131 isolates suggest that factors such as evolutionary mechanisms and antimicrobial resistance likely contribute to the global dissemination of these strains [75,76] and the widespread presence of ST131 in both human and animal populations complicate efforts to control its transmission [63].

In the analysis of the dendrogram resulting from the Bayesian analysis of the sequences of the seven genes (Supplementary File S16), it was observed that isolates originating from the same farm and sample, such as 3A-3 and 3A-6, both from milk in farm A, exhibited a high degree of genetic similarity. In addition, certain isolates from different farms also displayed genetic similarity, as evidenced by isolates 16E-6 (from the sieve in farm E) and 4B2-6 (from bovine feces in farm B), as well as isolates 2B-7 (from water in farm B) and 4D3-6 (from bovine feces in farm D).

The clustering of isolates in the dendrogram was not necessarily linked to their sequence type (ST), as many isolates had unknown STs. However, isolates with different STs originating from the same farm exhibited considerable genetic similarity, exemplified by 12C-10 (from a bucket) and 18C-5 (from cheese forms). Conversely, isolates with the same ST but from different sources, such as 2B-7 and 4D3-6, also clustered together in the same clade.

## 4. Conclusions

This study identified *E. coli* at various steps of milk obtainment and raw milk fresh cheese production, encompassing strains such as EPEC, STEC, and ExPEC, along with phylogroups associated with pathogenic isolates, like B2, D, and F. Isolates displayed notable antimicrobial resistance and harbored resistance genes. Moreover, crucial serogroups and sequence types (STs) associated with pathogenic isolates were identified. Genetic and epidemiological analyses revealed a circulation of these isolates across different sampling sites. Importantly, the study underlines that potentially pathogenic and antimicrobial-resistant *E. coli* in raw milk and fresh cheese produced using raw milk originate from diverse contamination sources, posing a potential risk to public health.

## Data Availability

Raw data are all available in Supplementary Files.

## Supplementary Materials

The following supporting information can be downloaded at https://www.mdpi.com/article/10.3390/microorganisms12081739/s1: Supplementary File S1. Sequence of initiator oligonucleotides used for amplifying genes associated with STEC, EPEC, ETEC, ExPEC, EIEC, and EAEC groups, along with the fragment size acquired, annealing temperature, control samples, and references. Supplementary File S2. Primer sequences for the genes chuA, YjaA, TspE4C2, Acek/ArpA1, ArpAgpE, trpAgpC, and trpBA, together with the size of the amplification product and the positive control. Supplementary File S3. Primer sequences for the antimicrobial resistance genes, along with the amplification product size, annealing temperature, positive control, and their corresponding references. Supplementary File S4. Pools of antisera used for detecting somatic antigen (O). Supplementary File S5. Sequence of oligonucleotide primers used for the *adk*, *fumC*, *gyrB*, *icd*, *mdh*, *purA*, and *recA* genes, along with the size of the amplification product and their corresponding annealing temperatures. Supplementary File S6. Presence of virulence genes defining STEC, EPEC, ETEC, EIEC, EAEC, and ExPEC pathotypes in samples collected from five dairy farms in the northeastern São Paulo State. Supplementary File S7. Virulence gene profiles of isolates found in positive samples across the five dairy properties producing raw cheese in the northeastern São Paulo State, Brazil. Supplementary File S8. Phylogroups to which isolates from three *E. coli* collections belong: commensal isolates, potentially pathogenic, and ESBL/AmpC producers. These data pertain to dairy properties producing Frescal cheese in the northeastern São Paulo State. Supplementary File S9. Correlation between the virulence gene profile and the phylogroup of isolates within the collection of potentially pathogenic strains and samples obtained from five dairy farms producing Frescal cheese in the northeastern São Paulo State. Supplementary File S10. Percentage of *E. coli* isolates resistant to antimicrobials, collected from five distinct dairy properties producing Frescal cheese in the northeastern São Paulo State. Supplementary File S11. Dendrogram illustrating the genetic relationship among *E. coli* isolates obtained from five distinct dairy farms producing raw milk cheese in the Jaboticabal region of the northeastern São Paulo State, using XbaI restriction. Supplementary File S12. Presence of resistance genes from the β-lactam and streptomycin (STR) groups, identified in *E. coli* isolates from five separate dairy farms producing Frescal cheese in the Jaboticabal region of the northeastern São Paulo State. Supplementary File S13. Presence of resistance genes related to the tetracycline group (TET), nalidixic acid (NAL), ciprofloxacin (CIP), trimethoprim/sulfamethoxazole (SXT), and sulfisoxazole (FIS) in E. coli isolates from five distinct dairy farms producing Minas Frescal cheese in the Jaboticabal region of the northeastern São Paulo State. Supplementary File S14. Relationship between somatic antigens (O), presence of virulence genes, and phylogroups of 18 potentially pathogenic *E. coli* isolates sourced from five different dairy farms manufacturing Frescal cheese in the northeastern São Paulo State, Brazil. Supplementary File S15. Sequence types (STs), origin, presence of virulence genes, antimicrobial resistance, phylogenetic group, resistance genes, and serogroup of 18 *E. coli* isolates collected from five different dairy farms producing Frescal cheese in the Jaboticabal region. Supplementary File S16. Tree resulting from Bayesian analysis of the sequences of *the adk*, *fumC*, *gyrB*, *icd*, *mdh*, *purA*, *and recA* genes from *E. coli* isolates.
