# Supplementary material for: Epidemiology, Virulence and Antimicrobial Resistance of Escherichia coli Isolated from Small Brazilian Farms Producers of Raw Milk Fresh Cheese
Source: Microorganisms. 2024 Aug 22;12(8):1739. doi: 10.3390/microorganisms12081739 (PMC11357254; doi:10.3390/microorganisms12081739)
Supplement: Supplementary file 1 [file microorganisms-12-01739-s001.zip › SF10_jmf.pdf]

Supplementary File S10. Percentage of *E. coli* isolates resistant to antimicrobials, collected from five distinct dairy properties producing Frescal cheese in the northeastern São Paulo State.

E. coli origin		No. Of isolates	% Isolates resistent by Category[a], antimicrobial class[b] e antimicrobial[c]													
			Category I					Category II							Category III	
			FLQ		PEN/I	CPS		PEN	CPM	AMG			FOL		PHE	TET
			NAL	CIP	AMC	TIO	CRO	AMP	FOX	GEN	KAN	STR	SXT	FIS	CHL	TET
Commensal collection	Farm A	66	16,67	0	0	0	0	13,64	0	0	0	7,58	6,06	6,06	1,52	16,67
	Farm B	60	28,33	0	3,33	1,67	1,67	8,33	6,66	0	3,33	6,66	3,33	8,33	1,67	18,33
	Farm C	57	22,81	0	1,75	0	0	3,51	5,26	3,51	0	5,26	1,75	5,26	0	8,77
	Farm. D	63	28,57	0	0	1,59	0	11,11	1,59	0	0	3,17	6,35	6,35	6,35	7,94
	Farm E	57	5,26	1,75	0	0	0	14,04	0	0	0	5,26	5,26	8,77	3,51	3,51
Potentially pathogenic	Farm A	18	11,11	0	0	0	0	22,22	0	0	22,22	27,77	11,11	27,77	0	22,22
	Farm B	18	77,77	0	55,55	0	0	0	5,55	0	5,55	5,55	0	5,55	11,1	77,77
	Farm C	29	0	0	0	0	0	93,1	0	0	0	93,1	93,1	93,1	0	17,24
	Farm. D	5	0	0	0	0	0	0	0	0	0	0	0	0	0	0
	Farm E	3	0	0	0	0	0	33,33	0	0	0	33,33	33,33	33,33	0	33,33
ESBL/AmpC producers	Farm C	5	0	0	100	100	0	100	100	0	0	0	0	0	0	0

[a] Category of antimicrobials important to humans: (I) Very important, (II) Important, (III) Moderate importance [b] Antimicrobial classes: (FLQ) fluoroquinolones; (PEN / I) penicillin + β-Lactamases inhibitors; (CPS) cephalosporins (PEN) penicillin; (CPM) cefamycin; (AMG) aminoglycosides; (FOL) folate; (PHE) phenicols; (TET) tetracyclines.[C] Antimicrobials: NAL, nalidixic acid; CIP, ciprofloxacin; AMC, amoxicillin / clavulanic acid; UNCLE, ceftiofur; CRO, ceftriaxone; AMP, ampicillin; FOX, cefoxitin; GEN, gentamicin; KAN, kanamycina; STR, streptomycin; SXT, trimethoprim-sulfamethoxazole; FIS, sulfisoxazole; CHL, chloramphenicol; ETT, tetracycline.
