# Supplementary material for: Epidemiology, Virulence and Antimicrobial Resistance of Escherichia coli Isolated from Small Brazilian Farms Producers of Raw Milk Fresh Cheese
Source: Microorganisms. 2024 Aug 22;12(8):1739. doi: 10.3390/microorganisms12081739 (PMC11357254; doi:10.3390/microorganisms12081739)
Supplement: Supplementary file 1 [file microorganisms-12-01739-s001.zip › SF11_jmf.pdf]

**Supplementary File S11.** Dendrogram illustrating the genetic relationship among *E. coli* isolates obtained from five distinct dairy farms producing raw milk cheese in the Jaboticabal region of northeastern São Paulo State, using XbaI restriction.

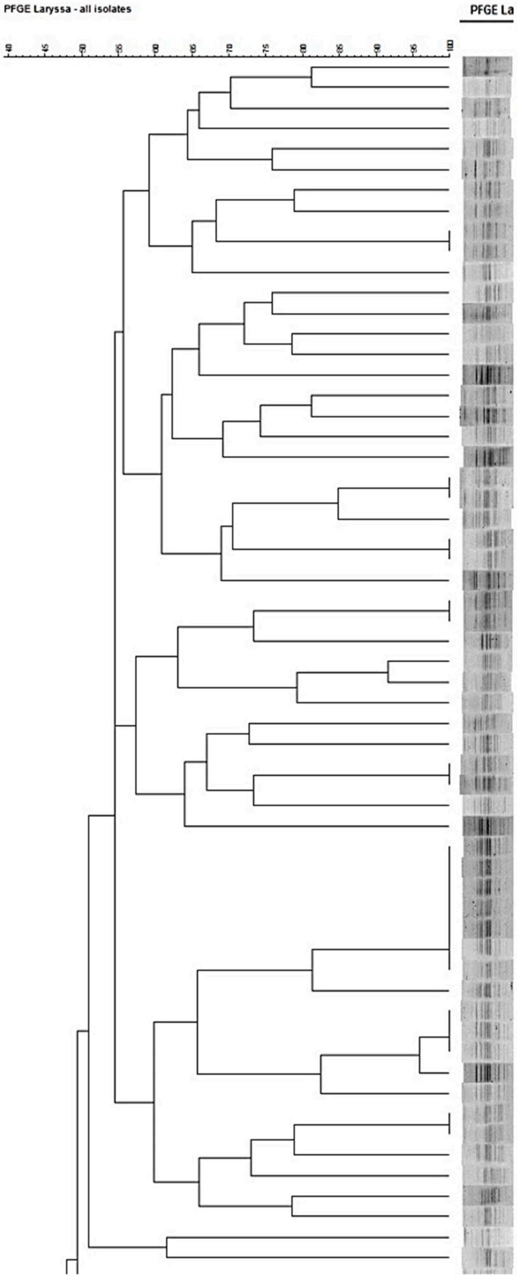


| Isolate | Farm | *E. coli group* | Virulence Gene | Phylogenetic group | Antimicrobial resistance | Gene (antimicrobial resistance) | Sample | Cluster | Serogroup |
|---|---|---|---|---|---|---|---|---|---|
| 19B-5 | B | - | - | A | NAL, STR, FIS, TET | tetA, qnrB | Dishcloth | 1 | |
| 3A-4 | A | - | - | A | AMP, STR, FIS, NAL, TET | blaSHV, tetA, aadA1, qnrB | Milk | 1 | |
| 4D3-7 | D | - | kps | A | - | - | Feces | 1 | O126 |
| 4aA-2 | A | - | - | A | - | - | Feces | 1 | |
| 18D-5 | D | - | - | B1 | - | - | Whey container | 1 | |
| 4D3-6 | D | - | kps | D | - | - | Feces | 1 | O7 |
| 13E-2 | E | - | - | A | - | - | Cheese moulds | 2 | |
| 8E-1 | E | - | - | A | - | - | Cheese producer man hands | 2 | |
| 10E-2 | E | - | - | B1 | - | - | Cheese | 2 | |
| 12E-1 | E | - | - | B1 | - | - | Sieve to filter milk | 2 | |
| 4dA-2 | A | - | - | A | TET | tetA | Feces | 2 | |
| 18C-2 | C | - | - | A | - | - | Cheese moulds | 3 | |
| 2C-1 | C | - | - | A | AMC, FOX, NAL | qnrB | Cheese preparation room water | 3 | |
| 10B-4 | B | - | - | A | - | - | Cheese | 3 | |
| 12D-2 | D | - | - | A | - | - | Sieve to filter milk | 3 | |
| 5B-4 | B | - | - | A | - | - | Milker man hands | 3 | |
| 3A-3 | A | - | iucD | B1 | AMP, STR, SXT, FIS, TET | blaTEM, tetA, dhfr V | Milk | 3 | O54 |
| 3A-6 | A | STEC | stx2 | B1 | - | - | Milk | 3 | O138 |
| 6C-1 | C | - | - | B1 | NAL | qnrB | Milk bucket | 3 | |
| 14B-2 | B | - | - | B1 | NAL | qnrB | Spoon | 3 | |
| 14A-12 | A | - | tsh | B1 | - | - | Bucket after heat milk | 3 | O18 |
| 14A-13 | A | - | tsh | B1 | KAN, NAL | qnrB | Bucket after heat milk | 3 | O18 |
| 14A-2 | A | - | - | B1 | AMP | - | Bucket after heat milk | 3 | |
| 10D-4 | D | - | - | B1 | - | - | Cheese | 3 | |
| 11D-4 | D | - | - | B1 | - | - | Cheese | 3 | |
| 4D4-5 | D | - | - | B1 | TIO, FOX, NAL | - | Feces | 3 | |
| 4B4-3 | B | - | - | A | AMP, TET | blaTEM, tetA | Feces | 4 | |
| 4B4-5 | B | - | - | A | AMP, TET | blaTEM, tetA | Feces | 4 | |
| 14E-1 | E | - | - | A | - | - | Sieve to filter milk | 4 | |
| 15E-5 | E | - | - | A | AMP, STR, SXT, FIS, TET | blaTEM, tetA | Bucket to heat milk | 4 | |
| 16E-6 | E | - | kps | A | AMP, STR, SXT, FIS, TET | blaTEM, tetA | Sieve to cheese production | 4 | O18 |
| 16E-4 | E | - | - | A | AMP, STR, SXT, FIS, TET | blaTEM, tetA | Sieve to cheese production | 4 | |
| 3E-5 | E | - | - | E | - | - | Milk | 5 | |
| 4E2-5 | E | - | - | B1 | NAL | - | Feces | 5 | |
| 3A-2 | A | ExPEC | iucD, PapC | B1 | AMP, KAN, STR, FIS, TET | blaTEM, tetA | Milk | 5 | O8 |
| 3A-7 | A | ExPEC | iucD, PapC | B1 | AMP, KAN, STR, FIS, TET | blaTEM, tetA | Milk | 5 | O8 |
| 18C-1 | C | - | - | B1 | - | - | Cheese moulds | 5 | |
| 7B-5 | B | - | - | B1 | AMC, KAN, STR, SXT, FIS, TET | - | Cheese elaboration surface | 5 | |
| 14B-4 | B | - | - | A | NAL | qnrB | Spoon | 6 | |
| 14D-2 | D | - | - | A | NAL | qnrB | Spoon | 6 | |
| 15D-1 | D | - | - | A | NAL | qnrB | Sieve to cheese production | 6 | |
| 16D-5 | D | - | - | A | NAL | qnrB | Cheese moulds | 6 | |
| 18D-3 | D | - | - | A | NAL | qnrB | Whey container | 6 | |
| 6C-2 | C | - | - | A | NAL | qnrB | Milk bucket | 6 | |
| 9D-1 | D | - | - | A | NAL | qnrB | Whey | 6 | |
| 15C-3 | C | - | - | A | NAL | qnrB | Thermometer | 6 | |
| 13C-2 | C | - | - | A | NAL | qnrB | Sieve to filter milk | 6 | |
| 5C-2 | C | - | - | A | NAL | qnrB | Milker man hands | 6 | |
| 8C-2 | C | - | - | A | NAL | qnrB | Cheese producer man hands | 6 | |
| 13B-1 | B | - | - | A | - | - | Bucket to heat milk | 6 | |
| 7D-4 | D | - | - | A | NAL, SXT, FIS, TET | tetA, qnrB | Cheese elaboration surface | 6 | |
| 10D-2 | D | - | - | A | - | - | Cheese | 6 | |
| 11D-2 | D | - | - | A | - | - | Cheese | 6 | |
| 7C-3 | C | - | - | A | NAL, TET | tetB, qnrB | Cheese elaboration surface | 6 | |
| 16C-2 | C | - | - | A | - | - | Spoon | 6 | |
| 2E-1 | E | - | - | A | - | - | Cheese preparation room water | 6 | |
| 4E4-1 | E | - | - | A | - | - | Feces | 6 | |
| 8A-2 | A | - | - | B1 | - | - | Cheese producer man hands | 7 | |
| 9C-1 | C | - | - | A | NAL | qnrB | Whey | 7 | |
| [illegible] | [illegible] | [illegible] | [illegible] | [illegible] | [illegible] | [illegible] | [illegible] | [illegible] | |

continues the dendrogram …

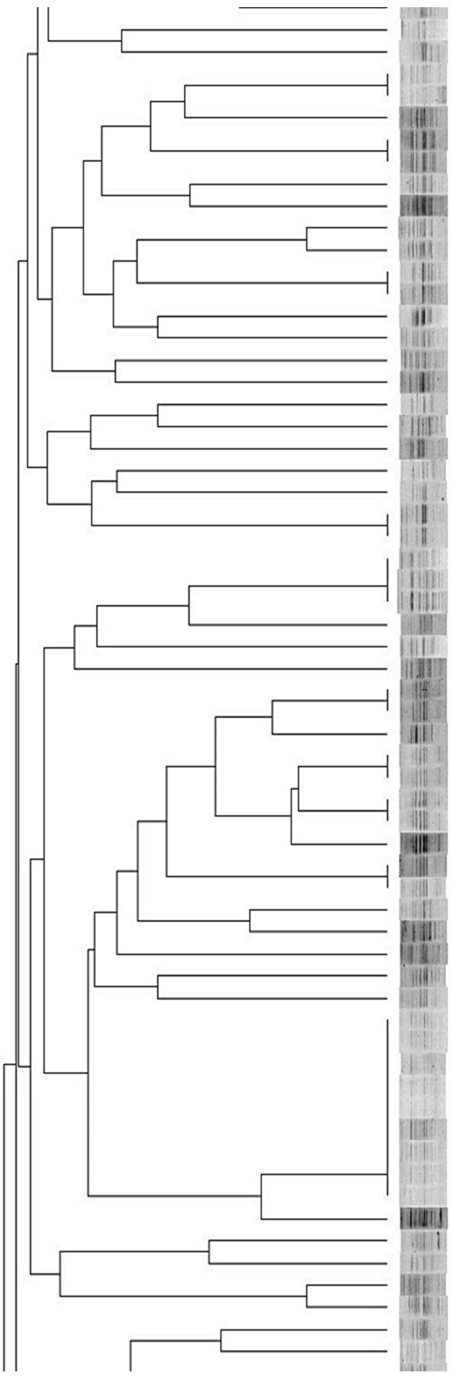

| | | | | | | | | | |
|---|---|---|---|---|---|---|---|---|---|
| [illegible] | [illegible] | - | - | [illegible] | - | - | [illegible] | [illegible] | |
| 8A-2 | A | - | - | B1 | - | - | Cheese producer man hands | 7 | |
| 9C-1 | C | - | - | A | NAL | qnrB | Whey | 7 | |
| 1A-1 | A | - | - | B1 | - | - | Milking parlor water | 8 | |
| 2A-5 | A | - | - | B1 | STR, SXT, NAL, TET | tetA, aadA1, dhfr I, qnrB | Cheese preparation room water | 8 | |
| 13D-4 | D | - | - | B1 | AMP | blaSHV | Bucket to heat milk | 8 | |
| 4D5-1 | D | - | - | B1 | - | - | Feces | 8 | |
| 6D-1 | D | - | - | B1 | - | - | Milk bucket | 8 | |
| 4E4-3 | E | - | - | B1 | AMP, SXT, FIS | blaTEM, dhfr V | Feces | 9 | |
| 6B-3 | B | - | - | B1 | NAL | qnrB | Liner from the milking machine | 9 | |
| 12C-3 | C | - | iucD | B1 | AMP | - | Milk bucket | 10 | |
| 15C-1 | C | - | - | B1 | - | - | Thermometer | 10 | |
| 5E-3 | E | - | - | B1 | - | - | Milker man hands | 10 | |
| 7E-2 | E | - | - | B1 | - | - | Cheese elaboration surface | 10 | |
| 4cA-3 | A | - | - | B1 | - | - | Feces | 10 | |
| 4E5-1 | E | - | - | A | - | - | Feces | 10 | |
| 13E-1 | E | - | - | B1 | AMP | blaSHV, blaCMY | Cheese moulds | 11 | |
| 14D-3 | D | - | - | B1 | - | - | Spoon | 11 | |
| 12D-4 | D | - | - | B1 | - | - | Sieve to filter milk | 12 | |
| 3D-6 | D | - | kps | D | - | - | Milk | 12 | O46 |
| 13D-2 | D | - | - | A | NAL, SXT, CHL, FIS, TET | tetA, qnrB | Bucket to heat milk | 13 | |
| 11C-6 | C | ExPEC | iucD, kps | U | TET | - | Cheese | 14 | O18 |
| 4D2-2 | D | - | - | B1 | - | - | Feces | 14 | |
| 4E1-2 | E | - | - | A | - | - | Feces | 15 | |
| 4E3-3 | E | - | - | A | - | - | Feces | 15 | |
| 2B-3 | B | - | kps | D | NAL, TET | qnrB | Cheese preparation room water | 16 | O18 |
| 2B-7 | B | - | kps | D | AMC, NAL, CHL, TET | qnrB | Cheese preparation room water | 16 | O18 |
| 2B-9 | B | - | kps | U | AMC, NAL, TET | qnrB | Cheese preparation room water | 16 | O18 |
| 5B-1 | B | - | - | B1 | - | - | Milker man hands | 16 | |
| 4dA-3 | A | - | - | B1 | - | - | Feces | 17 | |
| 4B3-1 | B | - | - | A | - | - | Feces | 18 | |
| 3B-2 | B | - | - | B1 | - | - | Milk | 19 | |
| 4B1-1 | B | - | - | B1 | TIO, CRO, FOX, KAN, NAL, STR,. | - | Feces | 19 | |
| 3E-1 | E | - | - | B1 | - | - | Milk | 19 | |
| 8D-1 | D | - | - | B1 | - | - | Cheese producer man hands | 19 | |
| 9D-3 | D | - | - | B1 | - | - | Whey | 19 | |
| 14C-3 | C | - | - | B1 | NAL | - | Bucket to heat milk | 19 | |
| 8C-3 | C | - | - | B1 | - | - | Cheese producer man hands | 19 | |
| 9B-3 | B | - | - | B1 | - | - | Whey | 19 | |
| 15B-2 | B | - | - | B1 | FOX | - | Cheese moulds | 19 | |
| 1B-3 | B | - | - | B1 | - | - | Milking parlor water | 19 | |
| 13C-3 | C | - | - | B1 | NAL | qnrB | Sieve to filter milk | 19 | |
| 17B-1 | B | - | - | B2 | - | - | Sieve to cheese production | 19 | |
| 4D1-2 | D | - | - | A | NAL, STR, SXT, CHL, FIS, TET | tetA, aadA1, qnrB | Feces | 19 | |
| 4C1-2 | C | - | - | B1 | - | - | Feces | 20 | |
| 4C1-3-CTX | C | - | - | B1 | AMC, TIO, AMP, FOX | blaCMY | Feces | 20 | |
| 11C-4 | C | - | - | B1 | AMP, STR, SXT, FIS | blaTEM, dhfr V | Cheese | 21 | |
| 12C-1 | C | - | iucD | B1 | AMP, STR, SXT, FIS, TET | blaTEM, dhfr V | Milk bucket | 21 | O117 |
| 12C-10 | C | - | iucD | B1 | AMP, STR, SXT, FIS | blaTEM, dhfr V | Milk bucket | 21 | O117 |
| 12C-16 | C | - | iucD | B1 | AMP, STR, SXT, FIS | blaTEM, dhfr V | Milk bucket | 21 | O117 |
| 17C-6 | C | - | iucD | B1 | AMP, STR, SXT, FIS | blaTEM, dhfr V | Sieve to cheese production | 21 | O117 |
| 17C-8 | C | - | iucD | U | AMP, STR, SXT, FIS | blaTEM, aadA1,dhfr V | Sieve to cheese production | 21 | O117 |
| 7C-6 | C | - | iucD | B1 | AMP, STR, SXT, FIS, TET | blaTEM, dhfr V | Cheese elaboration surface | 21 | O117 |
| 9C-6 | C | - | iucD | B1 | AMP, STR, SXT, FIS | blaTEM, dhfr V | Whey | 21 | O117 |
| 9C-8 | C | - | iucD | U | AMP, STR, SXT, FIS | blaTEM, dhfr V | Whey | 21 | O117 |
| 18C-5 | C | - | iucD | B1 | AMP, STR, SXT, FIS | blaTEM, dhfr V | Cheese moulds | 21 | O69 |
| 16E-3 | E | - | - | B1 | - | - | Sieve to cheese production | 22 | |
| 3D-1 | D | - | - | B1 | AMP | - | Milk | 22 | |
| 3C-2 | C | - | - | B1 | - | - | Milk | 23 | |
| 4C5-2 | C | - | - | B1 | - | - | Feces | 23 | |
| 16C-3 | C | - | - | B1 | - | - | Spoon | 24 | |
| 4bA-2 | A | - | - | B1 | AMP | blaSHV | Feces | 24 | |
| [illegible] | [illegible] | | | [illegible] | [illegible] | [illegible] | [illegible] | [illegible] | |

continues the dendrogram ….

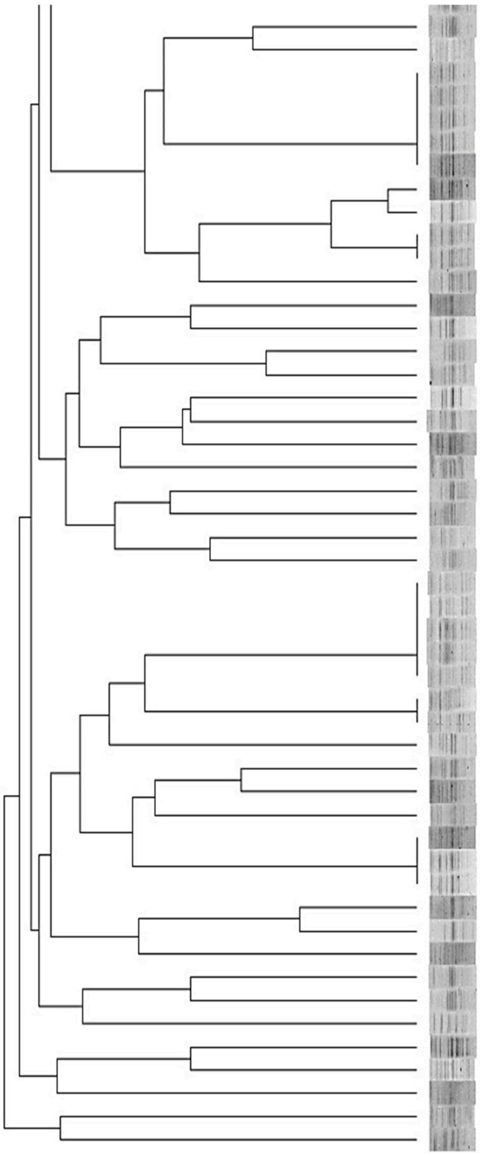

| | | | | | | | | | |
|---|---|---|---|---|---|---|---|---|---|
| [illegible] | [illegible] | | | [illegible] | | | [illegible] | [illegible] | |
| 16C-3 | C | - | - | B1 | - | - | Spoon | 24 | |
| 4bA-2 | A | - | - | B1 | AMP | blaSHV | Feces | 24 | |
| 11A-4 | A | - | - | B1 | STR, SXT, FIS, TET | tetA | Cheese | 24 | |
| 12A-3 | A | - | - | B1 | TET | tetC | Sieve to filter milk | 24 | |
| 13A-2 | A | - | - | B1 | - | - | Bucket before heat milk | 24 | |
| 17A-4 | A | - | - | B1 | - | - | Sieve to cheese production | 24 | |
| 18A-1 | A | - | - | B1 | - | - | Spoon | 24 | |
| 17B-3 | B | - | - | B1 | NAL | - | Sieve to cheese production | 24 | |
| 9A-1 | A | - | - | B1 | NAL | qnrB | Whey | 24 | |
| 15A-3 | A | - | - | B1 | NAL | qnrB | Tray | 24 | |
| 16A-5 | A | - | - | B1 | NAL | qnrB | Cheese moulds | 24 | |
| 7D-1 | D | - | - | B1 | NAL | - | Cheese elaboration surface | 24 | |
| 4D5-3 | D | - | - | A | - | - | Feces | 25 | |
| 4eA-2 | A | - | - | B1 | AMP | blaTEM | Feces | 25 | |
| 12B-4 | B | - | - | A | - | - | Milk bucket | 26 | |
| 1B-1 | B | - | - | A | - | - | Milking parlor water | 26 | |
| 10A-4 | A | - | - | B1 | TET | tetB | Cheese | 27 | |
| 4B2-6 | B | - | kps | A | TET | tetA | Feces | 27 | O69 |
| 4D3-2 | D | - | - | A | - | - | Feces | 27 | |
| 10D-6 | D | EPEC | eae, bfp | B1 | - | - | Cheese | 28 | O88 |
| 12B-2 | B | - | - | B1 | - | - | Milk bucket | 29 | |
| 4C4-5 | C | - | - | A | TET | tetC | Feces | 29 | |
| 2B-2 | B | - | - | A | - | - | Cheese preparation room water | 30 | |
| 2D-1 | D | - | - | A | AMP | - | Cheese preparation room water | 30 | |
| 6B-13 | B | - | kps | F | NAL, TET | qnrB | Liner from the milking machine | 31 | O71 |
| 6B-2 | B | - | kps | F | - | - | Liner from the milking machine | 31 | O71 |
| 6B-6 | B | - | kps | F | FOX, KAN, NAL, STR, FIS | qnrB | Liner from the milking machine | 31 | O71 |
| 6B-7 | B | - | kps | F | AMC, NAL, TET | qnrB | Liner from the milking machine | 31 | O71 |
| 6B-9 | B | - | kps | F | AMC, TET | - | Liner from the milking machine | 31 | O71 |
| 2B-4 | B | - | kps | B2 | NAL, TET | qnrB | Cheese preparation room water | 31 | O18 |
| 6B-8 | B | - | kps | B2 | AMC, NAL, TET | - | Liner from the milking machine | 31 | O18 |
| 11C-2 | C | - | - | A | TET | tetB | Cheese | 32 | |
| 11A-2 | A | - | - | A | - | - | Cheese | 33 | |
| 18B-3 | B | - | - | A | AMP, STR, FIS, TET | blaTEM, tetA | Cheese elaboration surface | 33 | |
| 9E-4 | E | - | - | A | - | - | Whey | 33 | |
| 5A-5 | A | - | - | B1 | STR, SXT, FIS, NAL, TET | aadA1, dhfr1, qnrB | Milker man hands | 34 | |
| 6A-1 | A | - | - | B1 | - | - | Milker bucket | 34 | |
| 7A-1 | A | - | - | B1 | - | - | Cheese elaboration surface | 34 | |
| 3E-4 | E | - | - | A | AMP, NAL, STR, CHL, FIS, TET | blaTEM, tetB, aadA1 | Milk | 35 | |
| 5E-4 | E | - | - | A | CIP, AMP, NAL, CHL, FIS, TET | blaTEM, tetB | Milker man hands | 35 | |
| 4B1-3 | B | - | - | E | - | - | Feces | 35 | |
| 10B-3 | B | - | - | B1 | AMC, TET | tetB | Cheese | 36 | |
| 17C-1 | C | - | - | A | GEN, STR, FIS | aadA1 | Sieve to cheese production | 36 | |
| 4E5-3 | E | - | kps | F | - | - | Feces | 37 | O83 |
| 11E-3 | E | - | - | B1 | - | - | Cheese | 38 | |
| 4aA-3 | A | - | - | B1 | TET | - | Feces | 38 | |
| 4B2-4 | B | - | - | A | NAL, SXT, FIS | qnrB | Feces | 39 | |
| 3E-6 | E | - | kps | A | - | - | Milk | 40 | O2 |
| 4C3-1 | C | - | - | A | - | - | Feces | 41 | |
