# Supplementary material for: Epidemiology, Virulence and Antimicrobial Resistance of Escherichia coli Isolated from Small Brazilian Farms Producers of Raw Milk Fresh Cheese
Source: Microorganisms. 2024 Aug 22;12(8):1739. doi: 10.3390/microorganisms12081739 (PMC11357254; doi:10.3390/microorganisms12081739)
Supplement: Supplementary file 1 [file microorganisms-12-01739-s001.zip › SF12_jmf.pdf]

Supplementary File S12. Presence of resistance genes from the β-lactam and streptomycin (STR) groups, identified in *E. coli* isolates from five separate dairy farms producing Frescal cheese in the Jaboticabal region of northeastern São Paulo State.

Collection		No. of resistant β-lactam isolates	Number (%) of β-lactam resistant isolates possessing β-lactamic genes					No. of STR resistant Isolates	Number (%) of STR-resistant isolates possessing *aadA1*gene	
			bla$_{CMY}$	*bla*$_{TEM}$	*bla*$_{SHV}$	*bla*$_{SHV}$: *bla*$_{CMY}$	None		*aadA*	None
Commensal collection	Farm A	4	0	1 (25)	2 (50)	0	1 (25)	4	3 (75)	1 (25)
	Farm B	7	0	3 (42,9)	0	0	4 (57,1)	4	1 (25)	3 (75)
	Farm C	3	0	1 (33,3)	0	0	2 (66,7)	2	1 (50)	1 (50)
	Farm. D	4	0	0	1 (25)	0	3 (75)	1	1 (100)	0
	Farm E	6	0	5 (83,3)	0	1 (16,7)	0	3	1 (33,3)	2 (66,7)
Potentially pathogenic	ExPEC	2	0	2 (100)	0	0	0	2	0	2 (100)
	STEC	0	0	0	0	0	0	0	0	0
	EPEC	0	0	0	0	0	0	0	0	0
	Potential EXPEC	17	0	11 (64,7)	0	0	6 (35,3)	13	0	13 (100)
Potential ESBL/AmpC	Farm C	1	1 (100)	0	0	0	0	0	0	0
