# Supplementary material for: Epidemiology, Virulence and Antimicrobial Resistance of Escherichia coli Isolated from Small Brazilian Farms Producers of Raw Milk Fresh Cheese
Source: Microorganisms. 2024 Aug 22;12(8):1739. doi: 10.3390/microorganisms12081739 (PMC11357254; doi:10.3390/microorganisms12081739)
Supplement: Supplementary file 1 [file microorganisms-12-01739-s001.zip › SF13_jmf.pdf]

Supplementary File S13. Presence of resistance genes related to the tetracycline group (TET), nalidixic acid (NAL), ciprofloxacin (CIP), trimethoprim-sulfamethoxazole (SXT), and sulfisoxazole (FIS), in E. coli isolates from five distinct dairy farms producing Minas Frescal cheese in the Jaboticabal region of northeastern São Paulo State.

Collection		No. of TET resistant isolates	Number (%) of TET resistant isolates possessing gene				No. of NAL and CIP resistant isolates	Number (%) NAL and CIP-resistant isolates possessing gene		No. of SXT and FIS resistant isolates	Number (%) of SXT and FIS-resistant isolates possessing gene		
			tetA	*tetB*	*tetC*	None		*qnrB*	None		*dfr 1*	*dfr 5*	None
Commensal collection	Farm A	8	4 (50)	1 (12,5)	1 (12,5)	2 (25)	7	7 (100)	0	3	2 (66,7)	1 (33,3)	0
	Farm B	7	5 (71,4)	2 (28,6)	0	0	8	6 (75)	2 (25)	5	1 (20)	0	4 (80)
	Farm C	3	0	2 (66,7)	1 (33,3)	0	11	10 (90,9)	1 (9,1)	2	0	1 (50)	1 (50)
	Farm. D	3	3 (100)	0	0	0	10	8 (80)	2 (20)	2	0	0	2 (100)
	Farm E	4	2 (50)	2 (50)	0	0	3	0	3 (100)	5	0	1 (20)	4 (80)
Potentially pathogenic	ExPEC	3	2 (66,7)	0	0	1 (33,3)	0	0	0	1	0	0	1 (100)
	STEC	0	0	0	0	0	0	0	0	0	0	0	0
	EPEC	0	0	0	0	0	0	0	0	0	0	0	0
	Potential ExPEC	13	3 (23,1)	0	0	10 (76,9)	10	10 (100)	0	13	0	10 (76,9)	3 (23,1)
Potential ESBL/AmpC	Farm C	0	0	0	0	0	0	0	0	0	0	0	0
