# Supplementary material for: Epidemiology, Virulence and Antimicrobial Resistance of Escherichia coli Isolated from Small Brazilian Farms Producers of Raw Milk Fresh Cheese
Source: Microorganisms. 2024 Aug 22;12(8):1739. doi: 10.3390/microorganisms12081739 (PMC11357254; doi:10.3390/microorganisms12081739)
Supplement: Supplementary file 1 [file microorganisms-12-01739-s001.zip › SF14_jmf.pdf]

Supplementary File S14. Relationship between somatic antigens (O), presence of virulence genes, and phylogroups of 18 potentially pathogenic *E. coli* isolates sourced from five different dairy farms manufacturing Frescal cheese in the northeastern São Paulo State Brazil.

O somatic antigen	Number of isolates:									
	iucD	*stx2*	*iucD:Papc*	*tsh*	*kps*				*iucD:kps*	*eae:bfp*
	B1	B1	B1	B1	A	B2	D	F	U	B1
O54	1	-	-	-	-	-	-	-	-	-
O138	-	1	-	-	-	-	-	-	-	-
O8	-	-	1	-	-	-	-	-	-	-
O18	-	-	-	1	1	1	1	-	1	-
O69	1	-	-	-	1	-	-	-	-	-
O71	-	-	-	-	-	-	-	1	-	-
O117	1	-	-	-	-	-	-	-	-	-
O46	-	-	-	-	-	-	1	-	-	-
O7	-	-	-	-	-	-	1	-	-	-
O126	-	-	-	-	-	1	-	-	-	-
O88	-	-	-	-	-	-	-	-	-	1
O2	-	-	-	-	1	-	-	-	-	-
O83	-	-	-	-	-	-	-	1	-	-
