# Supplementary material for: Epidemiology, Virulence and Antimicrobial Resistance of Escherichia coli Isolated from Small Brazilian Farms Producers of Raw Milk Fresh Cheese
Source: Microorganisms. 2024 Aug 22;12(8):1739. doi: 10.3390/microorganisms12081739 (PMC11357254; doi:10.3390/microorganisms12081739)
Supplement: Supplementary file 1 [file microorganisms-12-01739-s001.zip › SF15_jmf.pdf]

Supplementary File S15. Sequence types (ST), origin, presence of virulence genes, antimicrobial resistance, phylogenetic group, resistance genes, and serogroup of 18 *E. coli* isolates collected from five different dairy farms producing Frescal cheese in the Jaboticabal region.

Isolate	ST	Sample	Farm	Pathotype	Virulence gene	Antimicrobial resistance	Phylogroup	Resistant gene	Somatic antigen
10D-6	New ST	Cheese	D	EPEC	*eae, bfp*	-	B1	-	O88
11C-6	ST131	Cheese	C	ExPEC	*iucD, kps*	TET	U	-	O18
12C-10	ST58	Bucket	C		*iucD*	AMP, STR, SXT, FIS	B1	*blaTEM, dhfr V*	O117
14A-12	New ST	Bucket	A		*tsh*	-	B1	-	O18
16E- 6	New ST	Sieve	E		*kps*	AMP, STR, SXT, FIS, TET	A	*blaTEM, tetA*	O18
18C-5	ST5963	Cheese mold	C		*iucD*	AMP, STR, SXT, FIS	B1	*blaTEM, dhfr V*	O69
2B-7	ST38	Water form Cheese elaboration room	B		*kps*	AMC, NAL, CHL, TET	D	*qnrB*	O18
3A-3	New ST	Milk	A		*iucD*	AMP, STR, SXT, FIS, TET	B1	*blaTEM, tetA, dhfrV*	O54
3A-6	New ST	Milk	A	STEC	*stx2*	-	B1	-	O138
3A-7	New ST	Milk	A	ExPEC	*iucD, PapC*	AMP, KAN, STR, FIS, TET	B1	*blaTEM, tetA*	O8
3D-6	ST 349	Milk	D		*kps*	-	D	-	O46
3E -6	New ST	Milk	E		*kps*	-	A	-	O2
4B2 - 6	New ST	Bovine feces	B		*kps*	TET	A	*tetA*	O69
4D3-6	ST38	Bovine feces	D		*kps*	-	D	-	O7
4D3-7	New ST	Bovine feces	D		*kps*	-	A	-	O126
4E5-3	New ST	Bovine feces	E		*kps*	-	F	-	O83
6B-6	ST 5164	inner surface of the liner	B		*kps*	FOX, KAN, NAL, STR, FIS	F	*qnrB*	O71
6B-8	ST 676	inner surface of the liner	B		*kps*	AMC, NAL, TET	B2	-	O18
