# Supplementary material for: Epidemiology, Virulence and Antimicrobial Resistance of Escherichia coli Isolated from Small Brazilian Farms Producers of Raw Milk Fresh Cheese
Source: Microorganisms. 2024 Aug 22;12(8):1739. doi: 10.3390/microorganisms12081739 (PMC11357254; doi:10.3390/microorganisms12081739)

Supplementary File S16. Tree resulting from Bayesian analysis of the sequences of *the adk, fumC, gyrB, icd, mdh, purA, and recA* genes from *E. coli* isolates.

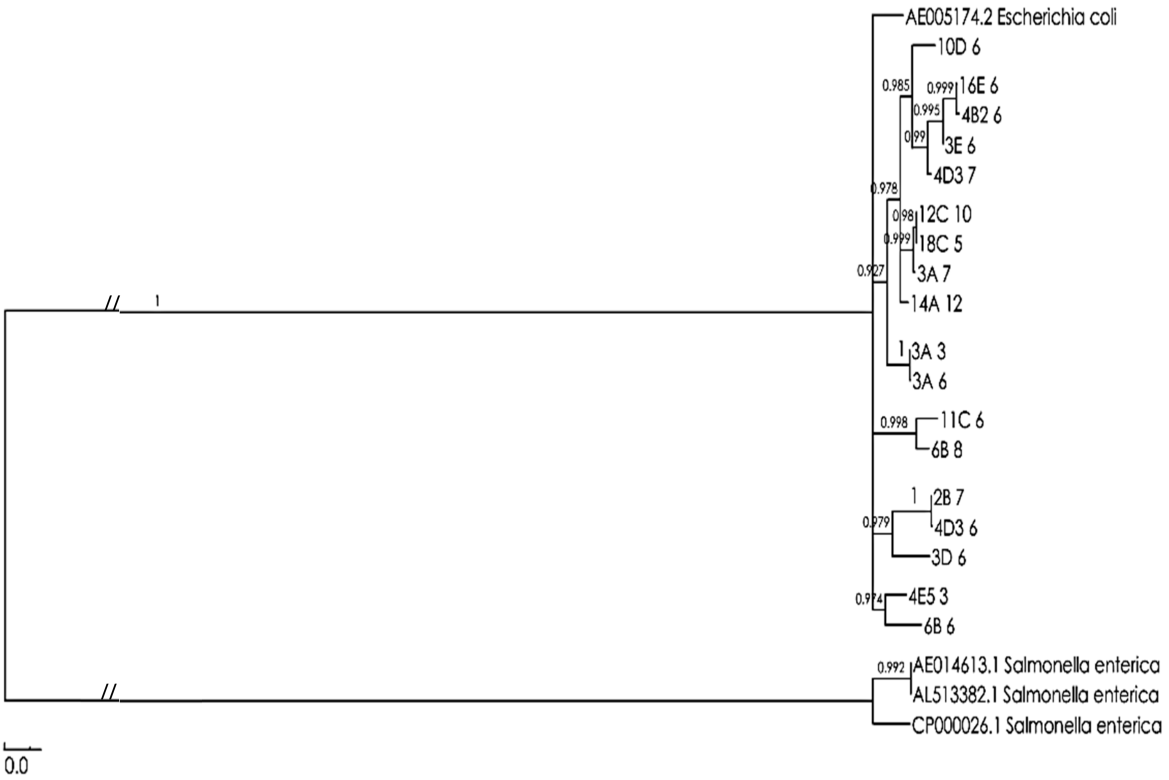

Supplement: Supplementary file 1 [file microorganisms-12-01739-s001.zip › SF16_jmf.pdf]
