# Supplementary material for: Epidemiology, Virulence and Antimicrobial Resistance of Escherichia coli Isolated from Small Brazilian Farms Producers of Raw Milk Fresh Cheese
Source: Microorganisms. 2024 Aug 22;12(8):1739. doi: 10.3390/microorganisms12081739 (PMC11357254; doi:10.3390/microorganisms12081739)
Supplement: Supplementary file 1 [file microorganisms-12-01739-s001.zip › SF1_jmf.pdf]

Supplementary File S1. Sequence of initiator oligonucleotides used for amplifying genes associated with STEC, EPEC, ETEC, ExPEC, EIEC, and EAEC groups, along with the fragment size acquired, annealing temperature, control samples, and references.

Pathotype	Virulence Factor	Gene	Sequence	Size (bp)	Pairing temperature (°C)	Positive control strain	Reference
STEC	Stx1	*stxA*	for 5' TTAGACTTCTCGACTGCAAAG<br>rev 5' TGTTGTACGAAATCCCCTCTG	530	60	EcL 6611	[12]
	Stx2all Shiga-like toxin type ll subunit A and B	*stx2A*	for 5' TTATATCTGCGCCGGGTCTG<br>rev 5' AGACGAAGATGGTCAAAACG	326			
EPEC	EAE (intimin)	*eae*	for 5' CATTATGGAACGGCAGAGGT<br>rev 5' ATCTTCTGCGTACTGCGTTCA	790			[13]
	Bundle forming pili fimbria	*bfp*	for 5' GGAAGTCAAATTCATGGGGGTAT<br>rev 5' GGAATCAGACGCAGACTGGTAGT	300	60	EcL2348/69	[14]
ETEC	STa	*estA*	for 5' TCCCCTCTTTTAGTCAGTCAACTG<br>rev 5' GCACAGGCAGGATTACAACAAAGT	163	60	EcL7805	[15]
	STb	*estB*	for 5' GCAATAAGGTTGAGGTGAT<br>rev 5' GCCTGCAGTGAGAAATGGAC	368			[16]
	LT	*eltB*	for 5' TTACGGCGTTACTATCCTCTCTA<br>rev 5' GGTCTCGGTCAGATATGTGATTC	275			[17]
	F4 K88ab1 and K88ab2	*faeG*	for 5' ATC GGT GGT AGT ATC ACT GC<br>rev 5' AAC CTG CGA CGT CAA CAA GA	601			[18]
ExPEC	CNF-1	*cnf*	for 5′ TCG TTA TAA AAT CAA ACA GTG<br>rev 5' CTT TAC AAT ATT GAC ATG CTG	633	55	EcL13421	[19]
	P fimbria	*papC*	for 5' TGA TAT CAC GCA GTC AGT AGC<br>rev 5' CCG GCC ATA TTC ACA TAA C	338			
	Aerobactin	*iucD*	for 5' AAGTGTCGATTTTATTGGTGTA<br>rev 5' CCATCCGATGTCAGTTTTCTG	778		EcL 3110	[20]
	temperature-sensitive hemagglutinin	*tsh*	for 5' GGTGGTGCACTGGAGTGG<br>rev 5' AGTCCAGCGTGATAGTGG	620			[21]
	S fimbriae adhesin	*sfa*	for 5' CTCCGGAGAACTGGGTGCATCTTAC<br>rev 5' CGGAGGAGTAATTACAAACCTGGCA	410	63	18147	[22]
	afimbrial adhesin VIII	*afa*	for 5'GGCAGAGGGCCGGCAACAGGC<br>rev 5' CCCGTAACGCGCCAGCATCTC	594		18162	
	protectins group II caps. polysacc. syn.	*kpsM II*	for 5'GCGCATTTGCTGATACTGTTG<br>rev 5' AGGTAGTTCAGACTCACACCT	570	63	O1:K7:H7	[22]
EIEC		*ipaH*	for 5' GTT CCT TGA CCG CCT TTC CGA TAC CGT C<br>rev 5' GCC GGT CAG CCA CCC TCT GAG AGT AC	620	60	H84-8226	[23]
		ial	for 5'CTG GAT GGT ATG GTG AGG<br>rev 5' GGA GGC CAA CAA TTA TTT CC	320			[24]
EAEC		*aaiC*	for 5' ATTGTCCTCAGGCATTTCAC<br>rev 5' ACGACAACCCCTGATAAACAA	215	60	17.2	[25]
		aatA	for 5' CTG GCG AAA GAC TGT ATC AT<br>rev 5' CAA TGT ATA GAA ATC CGC TGT T	629			[26]
		aggR	for 5' CTA ATT GTA CAA TCG ATG TA<br>rev 5' AGA GTC CAT CTC TTT GAT AAG	457			
