# Supplementary material for: Epidemiology, Virulence and Antimicrobial Resistance of Escherichia coli Isolated from Small Brazilian Farms Producers of Raw Milk Fresh Cheese
Source: Microorganisms. 2024 Aug 22;12(8):1739. doi: 10.3390/microorganisms12081739 (PMC11357254; doi:10.3390/microorganisms12081739)
Supplement: Supplementary file 1 [file microorganisms-12-01739-s001.zip › SF2_jmf.pdf]

Supplementary File S2. Primer sequences for the genes *chuA, YjaA, TspE4C2, Acek/ArpA1, ArpAgpE, trpAgpC*, and *trpBA*, together with the size of the amplification product and the positive control.

PCR reaction	Gene	Sequence	Size (bp)	Pairing temperature (°C)	Positive control
Quadruplex	*chuA*	for 5' ATGGTACCGGACGAACCAAC<br>rev 5' TGCCGCCAGTACCAAAGACA	288	59	ECOR70, EDL933, ECOR31, ECOR60
	yjaA	for 5' CAAACGTGAAGTGTCAGGAG<br>rev 5' AATGCGTTCCTCAACCTGTG	211		
	TspE4.C2	for 5' CACTATTCGTAAGGTCATCC<br>rev 5' AGTTTATCGCTGCGGGTCGC	152		
	arpAgpE	for 5' AACGCTATTCGCCAGCTTGC<br>rev 5' TCTCCCCATACCGTACGCTA	400		
Group E	*arpAgpC*	for 5' GATTCCATCTTGTCAAAATATGCC<br>rev 5' GAAAAGAAAAAGAATTCCCAAGAG	301	59	EDL933, ECOR31
Group C	*trpAgpC*	for 5' AGTTTTATGCCCAGTGCGAG<br>rev 5' TCTGCGCCGGTCACGCCC	219	57	ECOR70
Internal control	*trpBA*	for 5' CGGCGATAAAGACATCTTCAC<br>rev 5' GCAACGCGGCCTGGCGGAAG	489	-	-
