# Supplementary material for: Epidemiology, Virulence and Antimicrobial Resistance of Escherichia coli Isolated from Small Brazilian Farms Producers of Raw Milk Fresh Cheese
Source: Microorganisms. 2024 Aug 22;12(8):1739. doi: 10.3390/microorganisms12081739 (PMC11357254; doi:10.3390/microorganisms12081739)
Supplement: Supplementary file 1 [file microorganisms-12-01739-s001.zip › SF3_jmf.pdf]

Supplementary File S3. Primers sequences for the antimicrobial resistance genes, along with the amplification product size, annealing temperature, positive control, and their corresponding references.

Group	Gene	Sequence	Size (bp)	Pairing temperature (°C)	Positive control	Reference
Estreptomycin	*aadA1*	for 5' CATCATGAGGGAAGCGGTG<br>rev 5' GACTACCTTGGTGATCTCG	786	50	EcL 3482	[31]
Tetracycline	*tetA*	for 5' GTGAAACCCAACATACCCC<br>rev 5' GAAGGCAAGCAGGATGTAG	888	50	EcL 3482	
	tetB	for 5' CCTTATCATGCCAGTCTTGC<br>rev 5' ACTGCCGTTTTTTCGCC	774	55	EcL 12400	
Tetracycline	*tetC*	for 5' ACTTGGAGCCACTATCGAC<br>rev 5'CTACAATCCATGCCAACCC	881	50	PBR 322	[32]
Ceftiofur	*bla*$_{CMY-2}$	for 5' TGATGCAGGAGCAGGCTATTCC<br>rev 5' CTAACGTCATCGGGGATCTGC	323	63	EcL 3482	[33]
Ampicillin	*bla*$_{TEM}$	for 5' TTGCTCACCCAGAAACGCTGGTG<br>rev 5' TACGATACGGGAGGGCTTACC	708			
Ampicillin	*bla*$_{SHV}$	for 5' CGCCGGGTTATTCTTATTTGTCGC<br>rev 5' TCTTTCCGATGCCGCCGCCAGTCA	1016		PMON38	[34]
Ampicillin	*bla*$_{OXA}$	for 5' CGCAAATGGCACCAGATTCAAC<br>rev 5' TCCTGCACCAGTTTTCCCATACAG	464		EcL 12572	[33]
Ceftriaxone	*bla*$_{CTX-M}$	for 5' ATGTGCAGYACCAGTAARGTKATGGC<br>rev 5' TGGGTRAARTARGTSACCAGAAYCAGCGG	593		CTX-M-15	[33]
Trimethoprim-sulfamethoxazole	*dhfr I*	for 5' AAGAATGGAGTTATCGGGAATG<br>rev 5' GGGTAAAAACTGGCCTAAAATTG	391	50	EcL 3482	[35]
	dhfr V	for 5' CTGCAAAAGCGAAAAACGG<br>rev 5' AGCAATAGTTAATGTTTGAGCTAAAG	432	50	EcL 1329	
	dhfr VII	for 5' GGTAATGGCCCTGATATCCC<br>rev 5' TGTAGATTTGACCGCCACC	265	50	3B-0	
Quinolone (NAL, CIP)	*qnrB*	for 5' ACGATGCCTGGTAGTTGTCC<br>rev 5' ACGACATTCGTCAACTGCAA	469	53	298	[36]
