# Supplementary material for: Epidemiology, Virulence and Antimicrobial Resistance of Escherichia coli Isolated from Small Brazilian Farms Producers of Raw Milk Fresh Cheese
Source: Microorganisms. 2024 Aug 22;12(8):1739. doi: 10.3390/microorganisms12081739 (PMC11357254; doi:10.3390/microorganisms12081739)
Supplement: Supplementary file 1 [file microorganisms-12-01739-s001.zip › SF4_jmf.pdf]

Supplementary File S4. Pools of antisera used for detecting somatic antigen (O).

Pool	Antiserum
1	O8, 09, O20, O26, O64, O101, O141
2	O45, O138, O139, O147, O149, O157
3	O10, O108, O115, O119
1S	O1, O15, O21, O71, O83
2S	O2, O11, O18, O69, O131
3S	O4, O6, O22, O78, O137
4S	O53, O54, O55
5S	O35, O60, O73, O86
6S	O23, O34, O88, O111, O112, O120, O143
7S	O17, O49, O100, O117, O118, O123, O163
8S	O5, O27, 036, O76, O92
9S	O145, O148, O159, O173
10S	O46, O82, O84, O91, O98, O113
11S	O116, O121, 0126, O146, O171, O172
HUMAN	O25, O114, O125, 0
RABBIT 1	O7, O16, O75, O103
RABBIT 2	O109, O128, O132, O153
