# Supplementary material for: Epidemiology, Virulence and Antimicrobial Resistance of Escherichia coli Isolated from Small Brazilian Farms Producers of Raw Milk Fresh Cheese
Source: Microorganisms. 2024 Aug 22;12(8):1739. doi: 10.3390/microorganisms12081739 (PMC11357254; doi:10.3390/microorganisms12081739)
Supplement: Supplementary file 1 [file microorganisms-12-01739-s001.zip › SF5_jmf.pdf]

Supplementary File S5. Sequence of oligonucleotide primers used for *the adk, fumC, gyrB, icd, mdh, purA,* and *recA* genes, along with the size of the amplification product and their corresponding annealing temperatures.

Target	Sequence	Size (bp)	T (°C)
adk -F	ATTCTGCTTGGCGCTCCGGG	583	54
adk -R	CCGTCAACTTTCGCGTATTT		
fumC -F	TCACAGGTCGCCAGCGCTTC	806	54
fumC -R	GTACGCAGCGAAAAAGATTC		
gyrB -F	TCGGCGACACGGATGACGGC	911	60
gyrB -R	ATCAGGCCTTCACGCGCATC		
icd -F	ATGGAAAGTAAAGTAGTTGTTCCGGCACA	878	54
icd -R	GGACGCAGCAGGATCTGTT		
mdh -F	ATGAAAGTCGCAGTCCTCGGCGCTGCTGGCGG	932	60
mdh -R	TTAACGAACTCCTGCCCCAGAGCGATATCTTTCTT		
purA -F	CGCGCTGATGAAAGAGATGA	816	54
purA -R	CATACGGTAAGCCACGCAGA		
recA -F	CGCATTCGCTTTACCCTGACC	780	58
recA -R	AGCGTGAAGGTAAAACCTGTG		
