# Supplementary material for: Epidemiology, Virulence and Antimicrobial Resistance of Escherichia coli Isolated from Small Brazilian Farms Producers of Raw Milk Fresh Cheese
Source: Microorganisms. 2024 Aug 22;12(8):1739. doi: 10.3390/microorganisms12081739 (PMC11357254; doi:10.3390/microorganisms12081739)
Supplement: Supplementary file 1 [file microorganisms-12-01739-s001.zip › SF6_jmf.pdf]

Supplementary File S6. Presence of virulence genes defining STEC, EPEC, ETEC, EIEC, EAEC, and ExPEC pathotypes in samples collected from five dairy farms in the northeastern São Paulo State.

Farm	Sample	Pathotype	Virulence factor
A	Water (milk room)	EPEC	*eae*
	Milk	STEC, ExPEC	*stx2,iucD, PapC*
	Bovine feces	STEC	*stx2, CNF*
	Bovine feces	STEC	*stx2*
	Bovine feces	STEC	*stx2, kps*
	Bovine feces	STEC	*stx2*
	Sieve	ExPEC	*iucD, kps*
	Bucket	ExPEC	*tsh*
B	Water (cheese elaboration room)	Potentially ExPEC[a]	*kps*
	Bovine feces	Potentially ExPEC	*kps*
	Inner surface of liner	Potentially ExPEC	*kps*
C	Bovine feces	STEC, EPEC, ExPEC	*stx2, eae, iucD, PapC*
	Bucket	Potentially ExPEC	*kps*
	Cheese elaboration surface	Potentially ExPEC	*iucD*
	Serum	Potentially ExPEC	*iucD, kps*
	Cheese	Potentially ExPEC	*kps*
	Bucket	Potentially ExPEC	*iucD*
	Spoon	Potentially ExPEC	*iucD*
	Sieve	Potentially ExPEC	*iucD*
	Mold	STEC	*stx1, iucD*
D	Water (cheese elaboration room)	STEC	*stx2*
	Milk	Potentially ExPEC	*kps*
	Bovine feces	Potentially ExPEC	*kps*
	Cheese	EPEC	*eae*
E	Milk	Potentially ExPEC	*kps*
	Bovine feces	Potentially ExPEC	*tsh*
	Bovine feces	ExPEC	*tsh, kps*
	Sieve	Potentially ExPEC	*kps*
	Sieve	Potentially ExPEC	*kps*
