# Supplementary material for: Epidemiology, Virulence and Antimicrobial Resistance of Escherichia coli Isolated from Small Brazilian Farms Producers of Raw Milk Fresh Cheese
Source: Microorganisms. 2024 Aug 22;12(8):1739. doi: 10.3390/microorganisms12081739 (PMC11357254; doi:10.3390/microorganisms12081739)
Supplement: Supplementary file 1 [file microorganisms-12-01739-s001.zip › SF7_jmf.pdf]

Supplementary File S7. Virulence gene profiles of isolates found in positive samples across the five dairy properties producing raw cheese in the northeastern São Paulo State, Brazil.

Farm	Pathotype	Virulence gene profile	Sample (Number of Isolates)
A	ExPEC	*iucD:papC*	Milk (2)
	Potential ExPEC	*iucD*	Milk (2)
	STEC	*stx2*	Milk (1), Bovine feces (2)
	Potential ExPEC	*tsh*	Bucket (11)
B	Potential ExPEC	*kps*	Water (7), Bovine feces (1), liner (10)
C	Potentia ExPEC	*kps*	Whey (1)
	Potentia ExPEC	*iucD*	Cheese elaboration surface (1), Whey (5), Bucket (17), sieve (3), Mold (1)
	ExPEC	*iucD:kps*	Cheese (1)
D	Potential ExPEC	*kps*	Milk (2), Bovine feces (2)
	EPEC	*eae:bfp*	Cheese (1)
E	Potential ExPEC	*kps*	Milk (1), Bovine feces (1) sieve (1)
