# Supplementary material for: Epidemiology, Virulence and Antimicrobial Resistance of Escherichia coli Isolated from Small Brazilian Farms Producers of Raw Milk Fresh Cheese
Source: Microorganisms. 2024 Aug 22;12(8):1739. doi: 10.3390/microorganisms12081739 (PMC11357254; doi:10.3390/microorganisms12081739)
Supplement: Supplementary file 1 [file microorganisms-12-01739-s001.zip › SF8_jmf.pdf]

Supplementary File S8. Phylogroups to which isolates from three *E. coli* collections belong: commensal isolates, potentially pathogenic, and ESBL/AmpC producers. This data pertains to dairy properties producing Frescal cheese in the northeastern São Paulo State.

Phylogroup	No. Examined isolates	No. Isolates (Percentage)										
		Commensal isolate collection					Potentially pathogenics E. coli collection					ESBL/AmpC producer collection
		Farm A	Farm B	Farm C	Farm D	Farm E	Farm A	Farm B	Farm C	Farm D	Farm E	Farm. C
A	119	4 (6,06)	24 (40)	28 (49,12)	25 (39,68)	33 (57,89)	0	1 (5,55)	1 (3,45)	1 (20)	2 (66,66)	0
B1	224	60 (90,91)	31 (51,67)	27 (47,37)	35 (55,56)	22 (38,50)	18 (100)	0	25 (86,21)	1 (20)	0	5 (100)
B2	4	0	2 (3,33)	0	0	0		2 (10,53)	0	0	0	0
C	1	0	0	0	0	1 (1,75)		0	0	0	0	0
D	8	0	0	0	0	0		5(26,32)	0	3 (60)	0	0
E	2	0	1 (1,67)	0	0	1 (1,75)		0	0	0	0	0
F	10	0	0	0	0	0		9 (50,0)	0	0	1 (33,33)	0
Unknown	13	2 (3,03)	2 (3,33)	2 (3,51)	3 (4,76)	0		1 (5,55)	3 (10,34)	0	0	0
TOTAL	381	66	60	57	63	57	18	18	29	5	3	5
