# Supplementary material for: Epidemiology, Virulence and Antimicrobial Resistance of Escherichia coli Isolated from Small Brazilian Farms Producers of Raw Milk Fresh Cheese
Source: Microorganisms. 2024 Aug 22;12(8):1739. doi: 10.3390/microorganisms12081739 (PMC11357254; doi:10.3390/microorganisms12081739)
Supplement: Supplementary file 1 [file microorganisms-12-01739-s001.zip › SF9_jmf.pdf]

Supplementary File S9. Correlation between the virulence gene profile and the phylogroup of isolates within the collection of potentially pathogenic strains and samples obtained from five dairy farms producing Frescal cheese in the northeastern São Paulo State.

Farm	Sample	No. Of Isolates	Virulence gene profile	Phylogroup
A	Milk	2	*iucD:papC*	B1
		2	*iucD*	B1
		1	*stx2*	B1
	Bovine feces	2	*stx2*	B1
	Bucket	11	*tsh*	B1
B	Water from cheese elaboration room	5	*kps*	D
		1		B2
		1		U
	Bovine feces	1	*kps*	A
	Liner	9	*kps*	F
		1		B2
C	Cheese elaboration surface	1	*iucD*	B1
	Serum	4	*iucD*	B1
		1		U
		1	*kps*	A
	Bucket	17	*iucD*	B1
	Sieve	2	*iucD*	B1
		1		U
	Mold	1	*iucD*	B1
	Cheese	1	*iucD:kps*	U
D	Milk	2	*kps*	D
	Bovine feces	1	*kps*	D
		1		A
	Cheese	1	*eae, bfp*	B1
E	Milk	1	*kps*	A
	Bovine feces	1	*kps*	F
	Sieve	1	*kps*	A
